# *Helicobacter pylori* Perturbs Iron Trafficking in the Epithelium to Grow on the Cell Surface

**DOI:** 10.1371/journal.ppat.1002050

**Published:** 2011-05-12

**Authors:** Shumin Tan, Jennifer M. Noto, Judith Romero-Gallo, Richard M. Peek, Manuel R. Amieva

**Affiliations:** 1 Department of Microbiology and Immunology, Stanford University, Stanford, California, United States of America; 2 Department of Medicine, Vanderbilt University, Nashville, Tennessee, United States of America; 3 Department of Cancer Biology, Vanderbilt University, Nashville, Tennessee, United States of America; 4 Department of Pediatrics, Stanford University, Stanford, California, United States of America; Fred Hutchinson Cancer Research Center, United States of America

## Abstract

*Helicobacter pylori* (*Hp*) injects the CagA effector protein into host epithelial cells and induces growth factor-like signaling, perturbs cell-cell junctions, and alters host cell polarity. This enables *Hp* to grow as microcolonies adhered to the host cell surface even in conditions that do not support growth of free-swimming bacteria. We hypothesized that CagA alters host cell physiology to allow *Hp* to obtain specific nutrients from or across the epithelial barrier. Using a polarized epithelium model system, we find that isogenic Δ*cagA* mutants are defective in cell surface microcolony formation, but exogenous addition of iron to the apical medium partially rescues this defect, suggesting that one of CagA's effects on host cells is to facilitate iron acquisition from the host. *Hp* adhered to the apical epithelial surface increase basolateral uptake of transferrin and induce its transcytosis in a CagA-dependent manner. Both CagA and VacA contribute to the perturbation of transferrin recycling, since VacA is involved in apical mislocalization of the transferrin receptor to sites of bacterial attachment. To determine if the transferrin recycling pathway is involved in *Hp* colonization of the cell surface, we silenced transferrin receptor expression during infection. This resulted in a reduced ability of *Hp* to colonize the polarized epithelium. To test whether CagA is important in promoting iron acquisition *in vivo*, we compared colonization of *Hp* in iron-replete vs. iron-deficient Mongolian gerbils. While wild type *Hp* and Δ*cagA* mutants colonized iron-replete gerbils at similar levels, Δ*cagA* mutants are markedly impaired in colonizing iron-deficient gerbils. Our study indicates that CagA and VacA act in concert to usurp the polarized process of host cell iron uptake, allowing *Hp* to use the cell surface as a replicative niche.

## Author Summary

*Helicobacter pylori* (*Hp*) is a bacterium that chronically infects the stomach of humans and can lead to serious illness. To survive in the stomach, the bacteria intimately interact with the epithelial lining. Some inject the virulence protein CagA into the host cells, and we previously showed that CagA helps *Hp* survive and grow directly on the epithelial cell surface. Iron is one of the limiting factors that infectious bacteria must acquire from their host. Using a model polarized epithelium system, we discovered that CagA is able to alter the internalization, intracellular transport, and polarity of the transferrin/transferrin receptor iron uptake system. This allows the bacteria to shuttle iron across the epithelium and suggests a novel mechanism of iron acquisition from host cells, enabling *Hp* growth on the cell surface. Another major virulence factor of *Hp*, VacA, is also involved in this process. To test the role of CagA in iron acquisition *in vivo*, we infected iron-deficient Mongolian gerbils and found that CagA-deficient bacteria had a decreased ability to colonize the stomach. Our study illustrates how microbes that chronically infect our mucosal surfaces can manipulate the epithelium to acquire micronutrients from host cells and grow on the cell surface.

## Introduction

*Helicobacter pylori* (*Hp*) is a mucosal colonizer that infects the stomachs of more than half of the world's population [1]. Chronic *Hp* infection is a major cause of gastric and duodenal ulcer disease, a risk factor for gastric cancer [2], and recently has also been associated with iron deficiency anemia [3], [4]. During colonization of the stomach, a significant number of *Hp* (∼20%) adhere to the host cell surface via various adhesins [5]–[7]. We have previously reported that *Hp* can colonize and replicate directly while adhered to the epithelial surface, and can grow in this niche even in conditions where growth of the free-swimming bacteria is not supported [8]. The contact-dependent *Hp* virulence factor CagA, which is injected directly into host cells via the bacterium's type IV secretion system, plays an important role in enabling *Hp* colonization of the epithelium [8]. This occurs via a local perturbation of epithelial polarity, and can occur without gross disruption of epithelial integrity [8]. Since an important role of the epithelial barrier is to sequester and compartmentalize molecules that may be useful for colonizing microbes, we speculated that *Hp* has evolved specialized mechanisms to perturb cell polarity to acquire essential factors directly from the polarized epithelium. However, the nature of the factors transferred from the host cells to the bacteria and the molecular mechanisms involved remain unclear.

Successful colonization of mucosal surfaces by bacteria implies an ability to extract essential micronutrients from their immediate environment, either from epithelial secretions near the cell surface, from the polarized host cells themselves, and/or from the interstitial side, across the epithelial cell layer. Iron is a micronutrient critical for the survival and growth of many mucosal colonizers and its availability controls expression of bacterial virulence factors in *Hp* and several other pathogens [9]–[15]. In the host however, free iron exists in extremely limited quantities, since it is sequestered from the mucosal surface through various mechanisms, including the epithelial barrier blocking access to the interstitium, binding of interstitial iron by transferrin, sequestration of intracellular iron by ferritin, and chelation of mucosal iron by lactoferrin [13], [14]. While *Hp* is known to possess several iron uptake systems, the sources of iron that *Hp* utilizes during colonization of the gastric mucosa remain unclear [16]. Unlike other mucosal colonizers that possess siderophore-mediated mechanisms for uptake of iron [11], *Hp* has not been shown to synthesize siderophores [16]. While the acidity of the gastric lumen releases iron from ingested food [17], *Hp* is not found in the gastric lumen but rather colonizes the neutral environment of the epithelial cell surface and the overlying mucus layer [18]. In this microenvironment, iron is complexed with lactoferrin or with other glycoproteins found in the mucus [13], [19], [20]. *Hp* is unable to compete with partially saturated lactoferrin for iron acquisition [21], and its ability to obtain iron complexed with mucus glycoproteins is unknown. In the interstitium, iron is tightly bound to transferrin and *Hp* cannot compete with partially saturated transferrin for iron [21]. However, *Hp* is able to utilize iron from fully saturated transferrin [21], which is the major form endocytosed by epithelial cells [22].

In this study, we utilize a model polarized epithelium to show that *Hp* colonizing the apical surface are able to acquire iron from host epithelial cells. We also show that the *Hp* virulence factors CagA and VacA work in concert to shift the basolateral transferrin/transferrin receptor recycling process apically, directing transferrin and its receptor to sites of microcolony formation on the cell surface. Silencing expression of the transferrin receptor interferes with the colonization of the epithelium by *Hp*. Finally, we show, using a Mongolian gerbil model of *Hp* infection, that host iron depletion results in a decreased ability of CagA-deficient *Hp* to colonize the gastric niche.

## Results

### CagA facilitates iron acquisition during *Hp* microcolony growth on the apical cell surface

**Figure 1:**
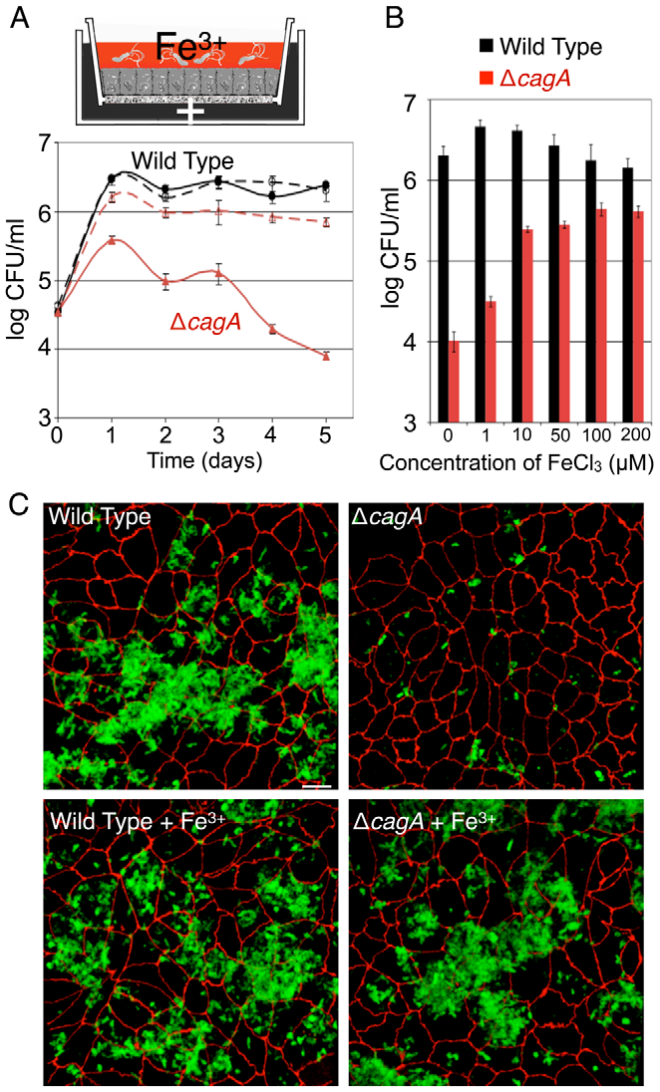
*Hp* acquires iron from host cells during colonization of the polarized epithelium. (A) Addition of iron allows Δ*cagA* growth on the apical cell surface. Polarized cells in the Transwell system were infected with WT or Δ*cagA*. Co-culture media (+) was added basally. Solid lines indicate conditions with DMEM apically. Dashed lines indicate conditions with 100 µM ferric chloride added to the apical DMEM. Samples were taken daily from the apical chamber and plated for CFU counts. (B) Δ*cagA* response to iron is concentration dependent. Polarized cells in the Transwell system were infected with WT or Δ*cagA*. Co-culture media was added basally, and different amounts of ferric chloride (FeCl_3_) in DMEM added to the apical chamber. Graph shows *Hp* CFU counts from the apical chamber at day 5 post-infection. (C) Exogenous iron rescues microcolony growth of Δ*cagA* on the cell surface. 3D confocal images of WT or Δ*cagA* colonizing the polarized epithelium 5 days post-infection, in the absence or presence of 100 µM ferric chloride (Fe^3+^). Bacteria are visualized with anti-*Hp* antibodies (green) and cell junctions are stained red (anti-ZO-1). Scale bar 10 µm.

We previously reported that wild type (WT) *Hp* are able to colonize the apical cell surface of polarized MDCK monolayers when the apical medium bathing the cells contains only DMEM, a medium that cannot support *Hp* growth *in vitro* [8]. However, CagA-deficient *Hp* (Δ*cagA*) are defective in colonizing this niche. This suggested that we could use this system to identify host factors obtained by WT *Hp* but unavailable to Δ*cagA* by enriching the apical medium with specific nutrients and testing which would rescue the growth defect of Δ*cagA*. We found that addition of iron in the form of ferric chloride to the apical chamber partially rescued the growth defect of Δ*cagA* in a saturable and dose dependent manner (Figure 1). In contrast, exogenous iron in the apical chamber of monolayers infected with WT did not lead to increased growth of the bacteria, indicating that rescue of the mutant is not due to a general enhancement of growth by a mechanism unrelated to CagA (Figures 1A and 1B). Imaging of bacteria adhered to the polarized monolayer revealed that iron added to the apical chamber enabled the growth of Δ*cagA* microcolonies adhered to the epithelium (Figure 1C). In addition, iron added to DMEM was not sufficient to sustain *Hp in vitro* (Figure S1A), suggesting that *Hp* colonizing the cell surface obtain not just iron, but also other essential factors through interaction with host cells.

The rescue of Δ*cagA* growing on the cell surface by iron suggests that CagA affects host epithelial cell function to allow *Hp* access to micronutrients that are found in the epithelium or across its barrier.

### *Hp* utilizes the polarized epithelium as a filter for the extraction of iron

**Figure 2:**
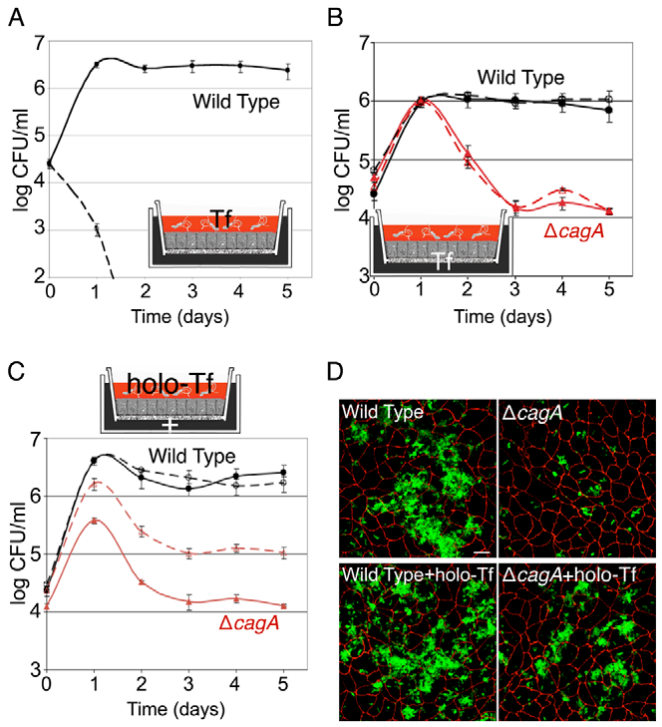
Holotransferrin promotes *Hp* microcolony growth on the cell surface. (A) Partially saturated transferrin inhibits *Hp* colonization of the apical cell surface. Polarized cells in the Transwell system were infected with WT. Co-culture media was added basally. Solid line indicates conditions where DMEM was present apically. Dashed line represents conditions where 75 µg/ml partially saturated transferrin (Tf) was added to the apical DMEM. Samples were taken daily from the apical chamber and plated for CFU counts. (B) Apical *Hp* microcolonies are protected by the epithelium from partially saturated transferrin in the basal chamber. Polarized cells in the Transwell system were infected with WT or Δ*cagA*. Solid lines indicate conditions where DMEM was present apically and co-culture media basally. Dashed lines represent conditions where DMEM was present apically and 75 µg/ml partially saturated transferrin (Tf) added to the co-culture media basally. Samples were taken and plated as in (A). (C) Holotransferrin partially rescues Δ*cagA* growth on the apical cell surface. Polarized cells in the Transwell system were infected with WT or Δ*cagA*. Co-culture media (+) was added basally. Solid lines indicate conditions with DMEM apically. Dashed lines indicate conditions with 75 µg/ml holotransferrin (holo-Tf) added to the apical DMEM. Samples were taken and plated as in (A). (D) Δ*cagA* forms microcolonies on the apical cell surface in the presence of holotransferrin. 3D confocal images of WT or Δ*cagA* colonizing the polarized epithelium 5 days post-infection, in the absence or presence of 75 µg/ml holotransferrin (holo-Tf) in the apical media. Bacteria are visualized with anti-*Hp* antibodies (green) and cell junctions are stained red (anti-ZO-1). Scale bar 10 µm.

Host epithelial cells acquire iron largely via transferrin receptor-mediated endocytosis [22]. In the serum where transferrin is normally found, the population of transferrin molecules is 20% – 40% saturated with iron [23], [24]. We thus wondered whether *Hp* growing on the cell surface gain access to interstitial transferrin and utilize this form of iron for survival. However, *in vitro* reports have shown that *Hp* cannot grow on partially saturated transferrin [21]. We found that *Hp* growth on the cell surface was inhibited by the presence of partially saturated transferrin in the apical medium, indicating that *Hp* is unable to compete with partially saturated transferrin for iron even in the presence of cells (Figure 2A).

Since *Hp* is susceptible to iron chelation by transferrin, and because transferrin is the major chelator of iron in extracellular fluids of the interstitial space, these results suggest that CagA's mechanism of action is not simply to injure the epithelium and provide paracellular diffusion of transferrin. We wondered if, instead, *Hp* utilizes the epithelial barrier as a shield from noxious macromolecules in the interstitial space, and whether the bacteria can actively obtain nutrients from the epithelium. To test this, we added transferrin to the basolateral co-culture medium at a concentration sufficient to chelate serum iron and inhibit *Hp* growth in broth (Figure S1B), and determined if the basal transferrin would inhibit growth of the apical bacteria. As shown in Figure 2B, transferrin at a concentration that quickly kills *Hp* in the apical chamber had no inhibitory effect across the polarized epithelium, indicating that *Hp* interaction with the apical cell surface allows the bacteria to utilize the epithelium as a filter for iron.

Epithelial cells selectively acquire transferrin saturated with iron (holotransferrin) via endocytosis, as its affinity for the transferrin receptor is 2000X greater than iron-free transferrin [22]. Since *Hp* growing on the cell surface are protected from the toxic effects of partially saturated transferrin by the epithelial barrier, we wondered whether the bacteria can obtain iron from holotransferrin, the form of transferrin taken up into cells. A recent report suggested that, in a chemically defined *in vitro* media system, *Hp* can obtain iron from holotransferrin, but not partially saturated transferrin [21]. We confirmed these results and tested whether holotransferrin affected the growth of *Hp* microcolonies on the cell surface by adding it to the apical medium of the Transwell culture system. Not only was holotransferrin not toxic to WT microcolonies, addition of holotransferrin to the apical chamber in fact led to partial rescue of Δ*cagA* (Figures 2C and 2D). These experiments indicate that *Hp* growing on the cell surface are able to utilize iron from holotransferrin, even though they cannot compete with partially saturated transferrin for iron, and suggest the hypothesis that acquisition of holotransferrin from within host cells is one mechanism by which *Hp* acquire iron during mucosal colonization.

### *Hp* colonization of the apical cell surface increases internalized transferrin in a CagA-dependent manner

Since CagA has multiple effects on epithelial physiology and appears to aid *Hp* in iron acquisition from host cells, we asked if *Hp* colonization of the apical cell surface affects host cell transferrin recycling. To study this, we utilized MDCK cells stably expressing human transferrin receptor [25], as this allowed us to visualize and quantify transferrin binding to its receptor and its uptake through the use of human transferrin conjugated to a fluorophore, which is readily available (Figure S2A). These cells stably expressing human transferrin receptor formed polarized monolayers, and WT *Hp* colonized the apical cell surface while Δ*cagA* exhibited a 100X defect in colonization ability, as seen with untransfected MDCK cells (Figure S2B).

**Figure 3:**
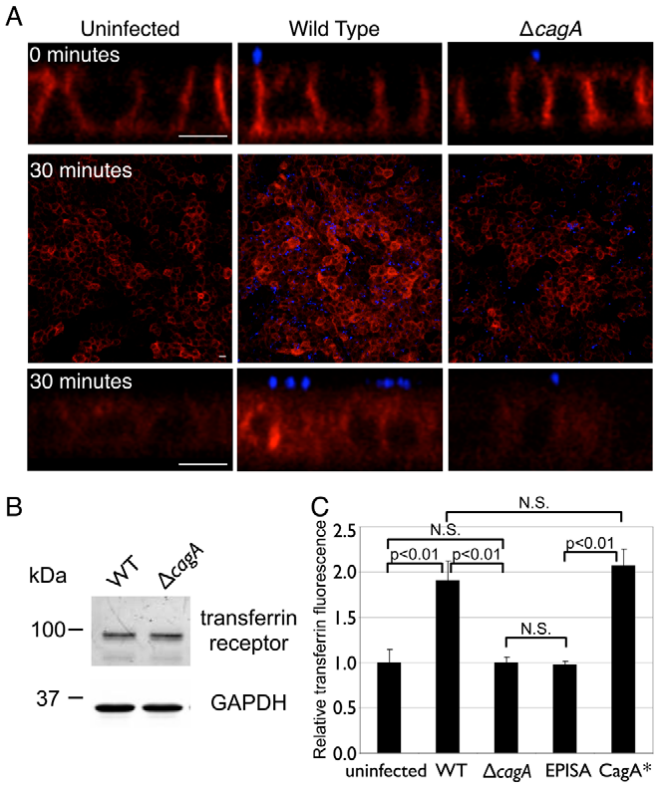
*Hp* colonization of the apical cell surface increases internalized transferrin. (A) 3D confocal images of fluorescent transferrin signal in polarized MDCK cells stably expressing human transferrin receptor, uninfected or infected with WT or Δ*cagA*. Fluorescent transferrin was added to the basal chamber and incubated on ice for 30 minutes, unbound transferrin washed away, then immediately fixed (top panels, cross-sectional view, 0 minutes post-uptake), or further incubated for 30 minutes at 37°C to allow uptake of bound transferrin (middle and bottom panels, top and cross-sectional views, 30 minutes post-uptake). Fluorescent transferrin is shown in red, and bacteria are visualized with anti-*Hp* antibodies (blue). Scale bars 10 µm. (B) *Hp* colonization does not significantly affect host cell transferrin receptor expression. Polarized cells in the Transwell system were infected for 2 days with WT or Δ*cagA*. Whole-cell lysates from these infections were separated by SDS-PAGE, transferred to a nitrocellulose membrane, then immunoblotted with antibodies against transferrin receptor (top panel) and against GAPDH as a loading control (bottom panel). (C) Quantification of transferrin fluorescence 30 minutes post-uptake in polarized epithelial monolayers. Monolayers were infected for 2 days with the indicated *Hp* strains, fixed after 30 minutes of transferrin uptake, and total transferrin fluorescence measured from multiple 3D confocal images. EPISA is a mutant expressing a mutated CagA that cannot be phosphorylated. CagA* is the complemented Δ*cagA* mutant. p-values were obtained with a Mann-Whitney statistical test. N.S. indicates no statistical significance.

Polarized MDCK cells stably expressing human transferrin receptor were either left uninfected or infected with WT or Δ*cagA* for two days before fluorescent transferrin assays were performed. In this assay, internalization of transferrin was synchronized by first adding transferrin to the basal chamber on ice. This allowed basolateral binding of transferrin to its receptor, but inhibited its uptake. Unbound transferrin was subsequently washed away, and the cells were warmed to 37°C to allow endocytosis to proceed. As expected, endocytosis was inhibited on ice and transferrin bound to the basolateral membranes of the polarized cells (Figure 3A, top panels) [22], [25]. There was no significant difference in the amount of transferrin bound in WT vs. Δ*cagA*-infected monolayers (Figure 3A, top panels). Also, by immunoblot, the expression level of the transferrin receptor under the different conditions was similar (Figure 3B). However, when endocytosis was allowed to proceed at 37°C for 30 minutes, significantly higher amounts of transferrin were observed inside the WT-infected monolayers as compared to uninfected or Δ*cagA*-infected monolayers (Figures 3A and 3C). We found similar results in Caco-2 cells (human colon carcinoma cells) (Figure S3A), indicating that these results are recapitulated in multiple epithelial lines. To determine if this difference is due to CagA vs. the density of colonizing bacteria on the cell surface, we repeated this experiment while adding exogenous iron to the apical chamber to rescue the growth defect of Δ*cagA*. In these conditions, the numbers of WT and Δ*cagA* growing on the cell surface were similar, yet the same difference in internalized transferrin was observed (Figures S3B and S3C). Finally, complementation of Δ*cagA* with the *cagA* gene (CagA*) led to restoration of the phenotype of increased transferrin internalization on infection with the bacteria (Figure 3C). These experiments suggest that CagA delivery into host cells increases the amount of internalized transferrin.

Once injected into the host cell, CagA is tyrosine phosphorylated by host Src- and Abl-family tyrosine kinases at several repeated sites in the C-terminal end containing EPIYA motifs [26]–[28]. CagA then acts as an adaptor protein that stimulates signaling downstream of growth factor receptor tyrosine kinases [29]–[32]. Since growth factor signaling increases transferrin uptake [33], we studied whether CagA phosphorylation is necessary for its effects on transferrin internalization. We found that the ability of CagA to increase internalized transferrin depends on the presence of the EPIYA motifs, as infection of polarized monolayers with a mutant lacking these phosphorylation domains resembled Δ*cagA* infection (Figure 3C).

These results indicate that CagA injected by *Hp* microcolonies on the apical cell surface increases transferrin internalization through receptor tyrosine kinase-like signaling, and suggest that this leads to an increased availability of iron for the colonizing bacteria. However, under normal conditions, transferrin should not be released to the apical side of an epithelium, since its recycling is confined to the basolateral membrane where the receptor is exclusively found [22]. This suggests that infecting bacteria perturb not just uptake, but also localization of the transferrin/transferrin receptor complex.

### Transferrin receptor is mislocalized to sites of *Hp* microcolony growth at the apical cell surface

*Hp* is known to affect host cell polarity and intracellular trafficking [34]–[38], and our previous study showed that perturbation of host cell polarity is involved in enhancing colonization of the polarized epithelium [8]. We therefore wondered if *Hp* colonization might lead to mis-sorting of the transferrin receptor, and hence transferrin and possibly iron, to sites of bacterial microcolony growth on the apical surface.

**Figure 4:**
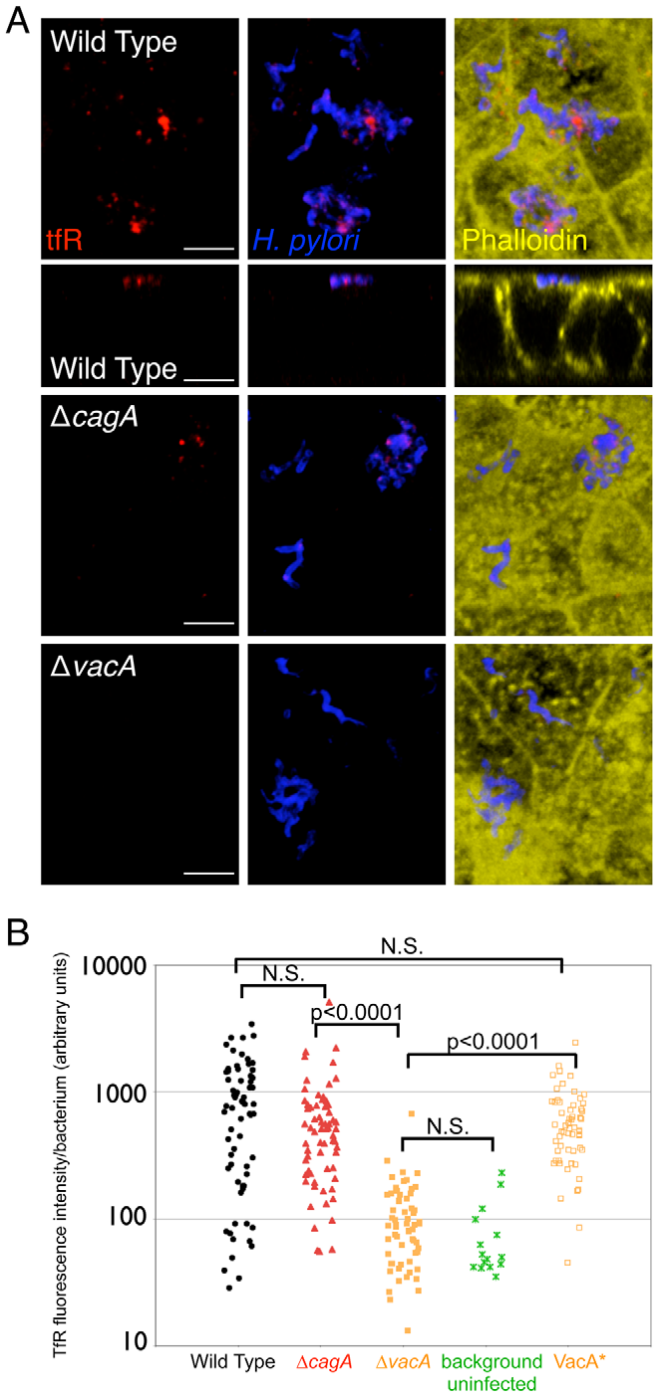
Transferrin receptor is mislocalized apically to sites of bacterial microcolonies. (A) Polarized MDCK cells in the Transwell system were infected with WT, Δ*cagA*, or Δ*vacA* for 2 days. Apical staining with anti-transferrin receptor antibodies was carried out on non-permeabilized samples. Bacteria are visualized with anti-*Hp* antibodies (blue), transferrin receptor (tfR) is stained red, and phalloidin staining of f-actin is shown in yellow. 3D confocal images are shown, and cross-sectional view is also presented for WT (second row). Scale bars 5 µm. (B) Quantitative data of the transferrin receptor (tfR) fluorescence intensity associated with the bacterial microcolonies, determined by the fluorescence voxel volume of each microcolony stained with anti-*Hp* antibodies, and the fluorescence sum of the transferrin receptor signal associated with the microcolonies, measured from multiple 3D confocal images. VacA* is the complemented Δ*vacA* mutant. Each point on the graph represents a microcolony. p-values were obtained with a Mann-Whitney statistical test. N.S. indicates no statistical significance.

To address this, we fixed uninfected or infected polarized MDCK monolayers in conditions that do not permeabilize the membrane and then applied antibodies against the transferrin receptor only to the apical side. In this manner, we can detect whether small amounts of transferrin receptor are found at the apical membrane without detecting internalized or basolateral transferrin receptor. We observed distinct puncta of immunolabeled transferrin receptor localized at the apical membrane near the *Hp* microcolonies (Figure 4A). This mislocalization of the transferrin receptor does not occur immediately after bacterial attachment to the apical surface, since monolayers fixed after 5 minutes of infection did not show puncta of transferrin receptor on the apical side (Figure S4A). This implies that *Hp* can affect host cell polarity locally to mislocalize basolateral proteins to sites of microcolony growth.

**Figure 5:**
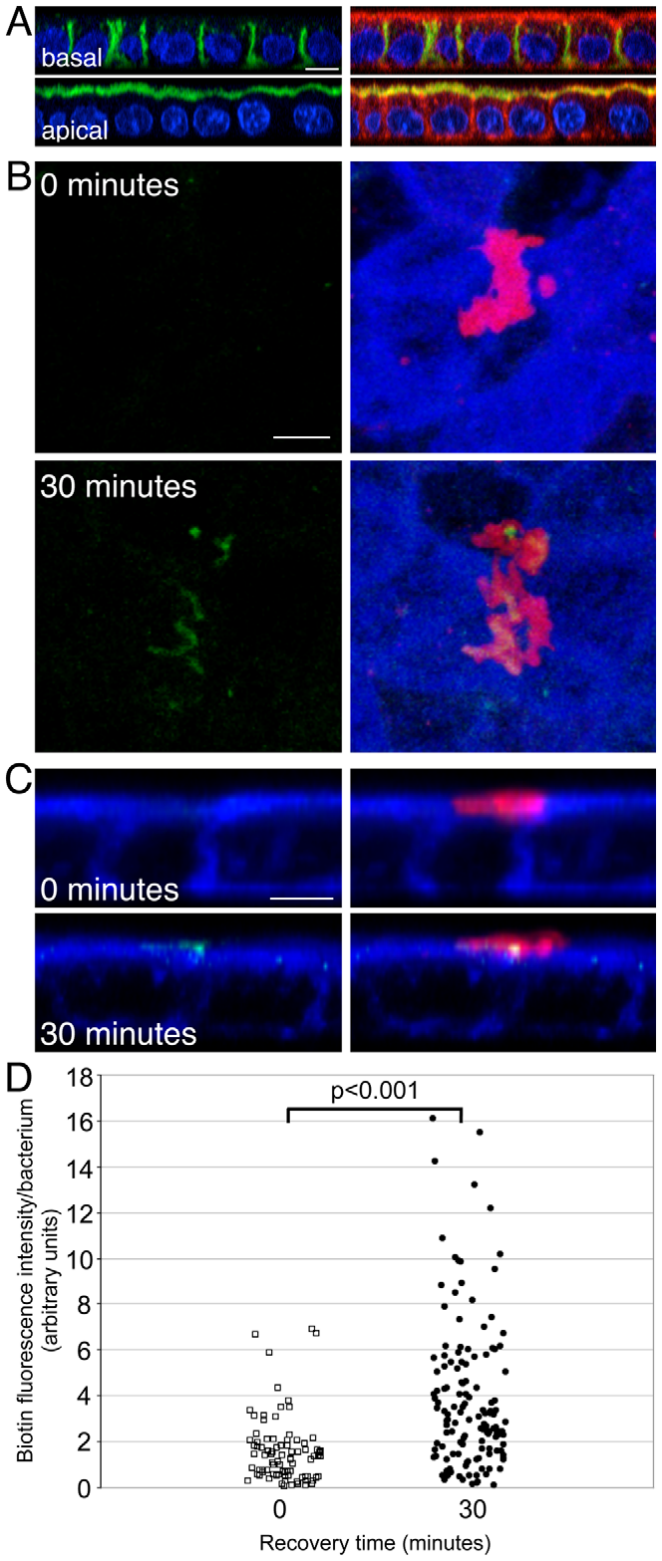
*Hp* induces mislocalization of basolateral proteins to the apical surface at sites of bacterial attachment. (A) Cell surface proteins of polarized cells on Transwell filters were selectively labeled with biotin in either the basal (top panels) or apical (bottom panels) chambers. Streptavidin labels biotinylated proteins in green, phalloidin stains f-actin in red and DAPI stains cell nuclei in blue. Scale bar 5 µm. (B and C) Polarized cells infected apically with WT for 2 days were selectively biotinylated on ice at the basolateral surface, and either immediately fixed (top panels, B and C), or first incubated for 30 minutes at 37°C (bottom panels, B and C) before fixation and apical streptavidin staining. Top views (B) and cross-sectional views (C) are shown. Bacteria are visualized with anti-*Hp* antibodies (red) and biotin is marked in green. Phalloidin staining of f-actin is shown in blue. Scale bars 5 µm. (D) Quantification of basolateral proteins associated with *Hp* microcolonies. Open squares are data from monolayers fixed immediately after basolateral biotinylation. Closed circles are data from monolayers incubated at 37°C for 30 minutes after basolateral biotinylation. Each point represents the total fluorescence intensity of apically-exposed biotin associated with a microcolony, divided by the number of bacteria present in that microcolony. p-value was obtained with a Mann-Whitney statistical test.

To confirm these findings, we used a different technique that allows selective biotinylation of surface proteins of the polarized epithelium (Figure 5A) [39], [40]. We selectively biotinylated the basolateral surface on ice, and then allowed internalization and recycling of the biotinylated basolateral proteins for 30 minutes at 37°C. We then stained the apical surface without permeabilization with fluorophore-conjugated streptavidin to detect mislocalized basolateral proteins. As with the previous results, we found that *Hp* microcolonies are associated with membrane patches containing basolateral proteins that were mis-sorted to the apical membrane (Figures 5B and 5C, bottom panels, and Figure 5D). To confirm that endocytosis is required, we repeated the staining on infected monolayers that were fixed immediately after surface biotinylation on ice. These monolayers did not contain apical biotin staining (Figures 5B and 5C, top panels, and Figure 5D). To determine whether this phenomenon is generalizable to other polarized cell models, we repeated the staining of transferrin receptor in Caco-2 cells. We obtained similar results as with MDCK cells, indicating that *Hp* can induce mis-sorting of the transferrin receptor in multiple epithelial lines (Figure S5). Finally, to determine whether mislocalization of basolateral proteins is restricted to a subset of proteins or affects all basolateral proteins, we used antibodies to other basolateral markers. E-cadherin is a cell-cell adhesion molecule that is normally absent from the apical membrane. Using apical staining of non-permeabilized cell monolayers with E-cadherin antibodies, we had previously shown that E-cadherin is absent from most of the apical membrane except for sites of cell extrusion [41]. When we applied these antibodies to *Hp*-infected monolayers, we did not find E-cadherin associated with *Hp* microcolonies (Figure S4B), indicating that not all basolateral proteins are mislocalized during *Hp* colonization.

Examination of monolayers infected with Δ*cagA* indicated that CagA-deficient bacteria are less efficient but still able to recruit transferrin receptor to the sites of bacterial microcolonies (Figure 4). Quantification of the amount of transferrin receptor observed apically at the sites of Δ*cagA* microcolonies showed that the median amount (514 arbitrary units/bacterium) was less than that observed with WT (774 arbitrary units/bacterium), but this was not statistically significant (Figure 4B).

The ability of Δ*cagA* to still cause mislocalization of host cell transferrin receptor apically to sites of bacterial microcolonies suggested that other bacterial factor(s) may be involved in this phenomenon. Another major virulence factor of *Hp*, vacuolating cytotoxin (VacA), has been reported to interfere with the endocytic pathway of host cells [38]. We therefore decided to test the role of VacA in the mislocalization of transferrin receptor. We constructed a VacA-deficient *Hp* mutant (Δ*vacA*), and found that in monolayers infected with Δ*vacA*, transferrin receptor was absent from the sites of bacterial microcolonies on the apical cell surface (Figure 4). This result was confirmed in infected Caco-2 cell polarized monolayers (Figure S5). We also complemented Δ*vacA* with the *vacA* gene, and found that this reconstitution (VacA*) restored the ability of the bacteria to mislocalize transferrin receptor apically to sites of bacterial microcolonies (Figure 4B).

Selective biotinylation of basolateral epithelial proteins and quantification of the amount of biotin subsequently associated with apical microcolonies indicated that both Δ*cagA* and Δ*vacA* had significantly less biotin associated with microcolonies than WT (Figure S6). This suggests that both CagA and VacA are involved in disruption of polarity, and that the transferrin receptor is not the only molecule that is mislocalized apically. We also found that a mutant deficient in both CagA and VacA (Δ*cagA*Δ*vacA*) was still able to mislocalize proteins to the sites of bacterial microcolonies (Figure S6), implying that CagA and VacA are not the only bacterial factors involved in this process, although they do appear to play major roles.

Together, these results indicate that *Hp* colonization of the apical cell surface leads to mislocalization of a subset of basolateral proteins to the apical cell membrane at sites of bacterial microcolony growth, with the *Hp* virulence factors CagA and VacA playing major roles in this process. One of the proteins mislocalized to these sites is the transferrin receptor, and its mislocalization is primarily dependent on VacA.

### VacA aids *Hp* colonization of the apical cell surface

**Figure 6:**
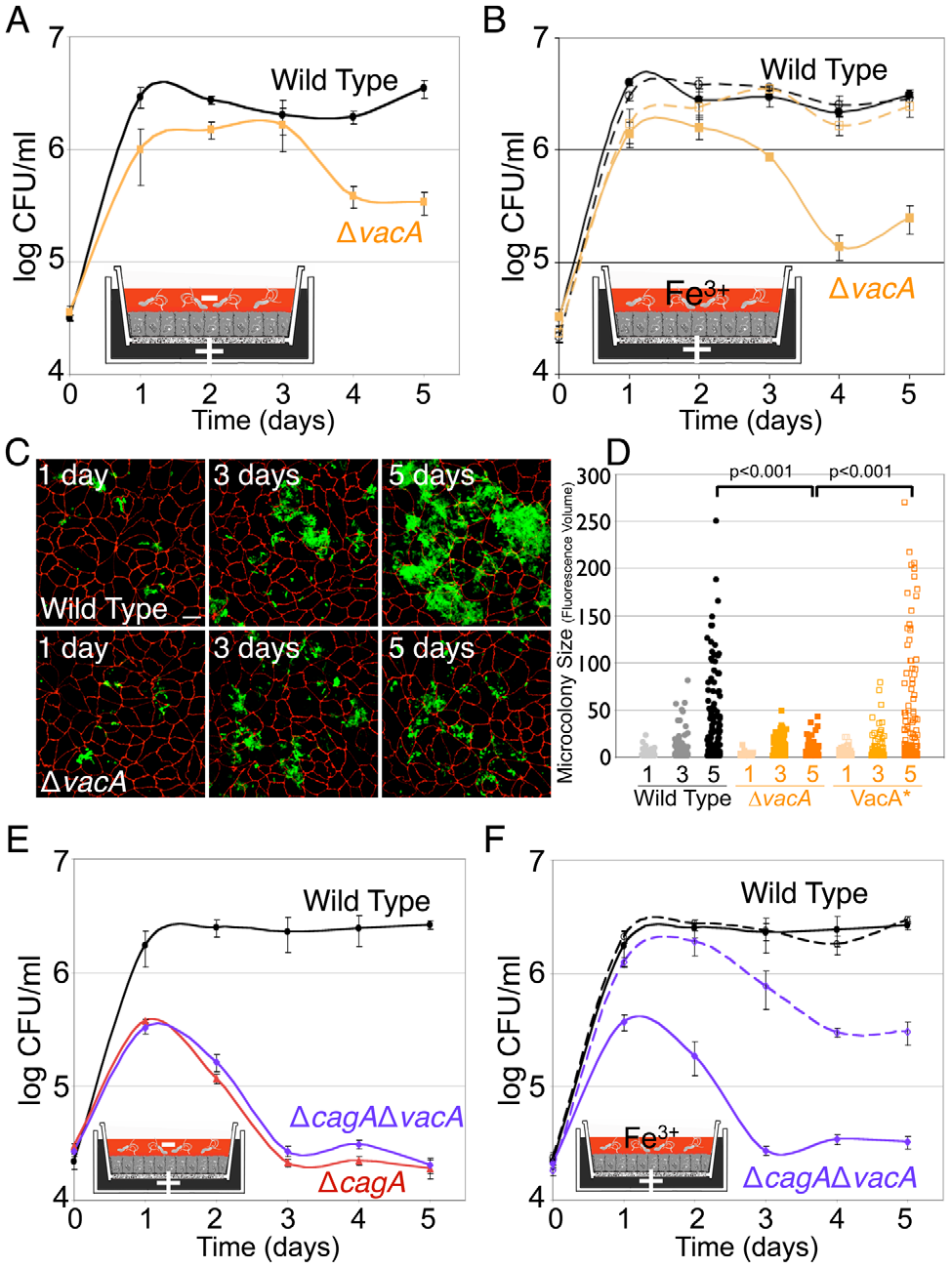
VacA contributes to *Hp* colonization of the apical cell surface. (A) VacA aids *Hp* colonization of the apical cell surface. Using the Transwell system, cells were infected with WT or Δ*vacA*, and co-culture media added only to the basal chamber (+). DMEM was added to the apical chamber (−). Samples were taken daily from the apical chamber and plated for CFU counts. (B) Exogenous addition of iron apically rescues Δ*vacA* growth on the polarized epithelium. Polarized cells were infected as in (A). Solid lines indicate conditions with DMEM apically. Dashed lines indicate conditions with 100 µM ferric chloride (Fe^3+^) added to the apical DMEM. Samples were taken and plated as in (A). (C) 3D confocal images of WT or Δ*vacA* colonizing the cell surface of polarized MDCK cells in the Transwell system. Cells were infected for 5 minutes and then unattached bacteria washed away and media replaced. At 1, 3 and 5 days post-infection, samples were fixed and processed for immunofluorescence. Bacteria are visualized with anti-*Hp* antibodies (green) and cell junctions are stained red (anti-ZO-1). Scale bar 10 µm. (D) Quantification of WT, Δ*vacA* and VacA* microcolony sizes over time (1, 3 and 5 days), determined by fluorescence volume measured from multiple 3D confocal images. VacA* is the complemented Δ*vacA* mutant. Each point on the graph represents a microcolony. p-value was obtained with a Mann-Whitney statistical test. (E) CagA and VacA work in concert to enable *Hp* colonization of the polarized epithelium. Polarized cells were infected as in (A) with the strains indicated. Samples were taken and plated as in (A). (F) CagA and VacA work together to aid *Hp* acquisition of iron from host cells. Polarized cells were infected as in (A). DMEM (solid lines) or DMEM + 100 µM ferric chloride (Fe^3+^, dashed lines) was added apically. Samples were taken and plated as in (A).

Given the role of VacA in transferrin receptor mislocalization, we next tested whether VacA affects the ability of *Hp* to colonize the apical cell surface of a polarized epithelium. We observed that Δ*vacA* have approximately a 10X decrease in bacterial counts at day 5 post-infection, as compared to WT (Figure 6A). In the presence of rich media in the apical chamber, Δ*vacA* grow as well as WT, indicating that this phenotype is not due to an *in vitro* growth defect of the mutant (Figure S7).

To determine whether the decrease in bacterial counts correlates with a defect of colonization of the apical cell surface, we examined microcolony formation on polarized cells infected with Δ*vacA* by confocal immunofluorescence microscopy. Initial adherence of Δ*vacA* to the apical cell surface was no different from WT, with an average of 9 bacteria adhered/100 cells in each case (p = 0.5). However, Δ*vacA* formed significantly smaller microcolonies on the cell surface at day 5 post-infection (Figures 6C and 6D). Complementation of Δ*vacA* (VacA*) led to restoration of the ability of the bacteria to effectively colonize the polarized epithelium, with the formation of large microcolonies (Figure 6D).

We also tested whether the Δ*vacA* mutant is defective in iron acquisition from the host, by supplementing the apical media with iron. Exogenous addition of iron to the apical chamber led to rescue of the colonization defect shown by Δ*vacA* (Figure 6B). To examine if CagA and VacA act in similar or different pathways in aiding *Hp* in cell surface colonization, we tested the mutant deficient in both CagA and VacA (Δ*cagA*Δ*vacA*). This double mutant resembled the single CagA-deficient mutant, and exogenous addition of iron apically partially rescued its ability to colonize the polarized epithelium (Figures 6E and 6F). The phenotype observed is not due to the mutant having an *in vitro* growth defect, as growth of this double mutant was similar to WT in the presence of rich media added to the apical chamber (Figure S7).

These results indicate a role for VacA in *Hp* colonization of the polarized epithelium, and suggest that CagA and VacA work in concert to aid *Hp* acquisition of iron from, and colonization of, host polarized epithelial cells.

### Host cell transferrin receptor is involved in *Hp* microcolony formation on the apical cell surface

The data presented above indicate that the *Hp* virulence factors CagA and VacA both affect normal recycling and trafficking of the transferrin/transferrin receptor complex, which is the major iron uptake mechanism of epithelial cells [22]. Furthermore, mutants in both virulence factors are defective in colonizing the polarized cell surface and these defects can be partially rescued by exogenous addition of iron. This suggests that *Hp* perturbation of the transferrin/transferrin receptor recycling pathway might be used by the bacteria to obtain iron from host cells.

**Figure 7:**
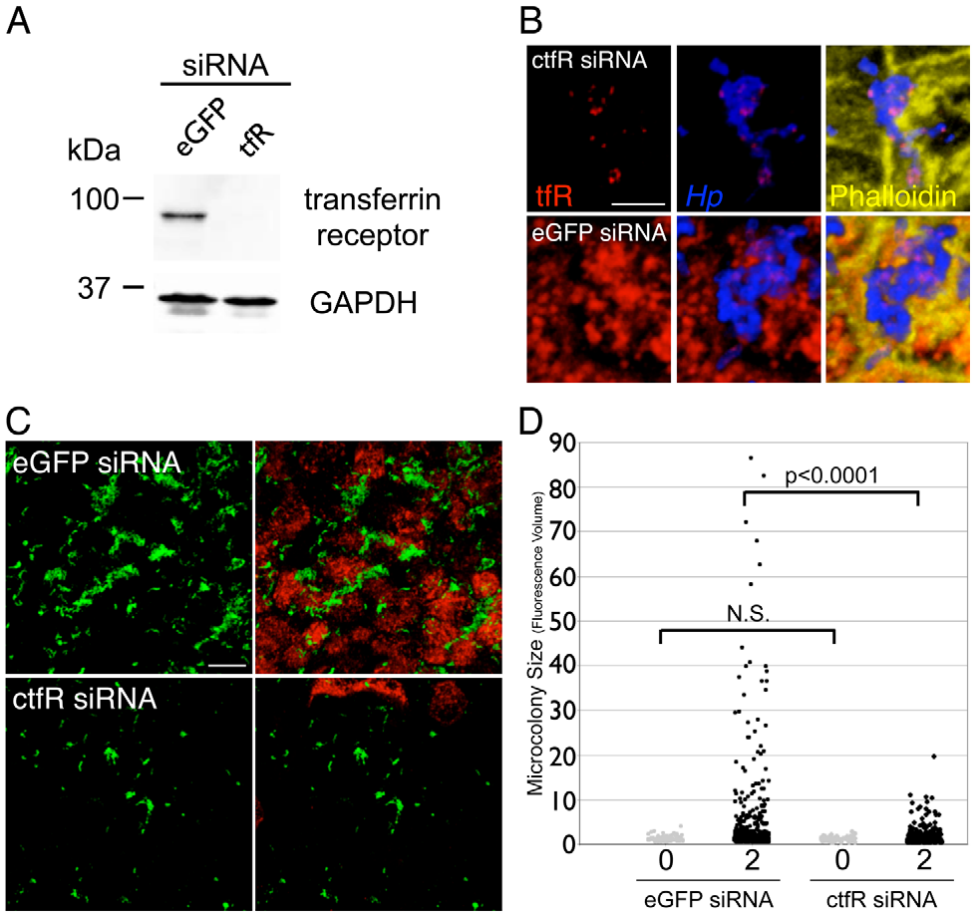
Down-regulation of host cell transferrin receptor decreases *Hp* microcolony growth on the cell surface. (A) siRNA knockdown of transferrin receptor expression in MDCK cells. Cells were transfected with a combination of two siRNAs directed against transferrin receptor, or with siRNA directed against enhanced GFP (eGFP) as a control. 3 days post-transfection, the cells were collected and lysates separated by SDS-PAGE, transferred to a nitrocellulose membrane, then immunoblotted with antibodies against transferrin receptor (top panel) and against GAPDH as a loading control (bottom panel). (B) Residual transferrin receptor is mislocalized apically to sites of bacterial microcolonies. MDCK cells were transfected with siRNA against canine transferrin receptor (ctfR, top panels) or eGFP as a control (bottom panels). After polarization, the cells were infected with WT for 1 day. Bacteria are visualized with anti-*Hp* antibodies (blue), transferrin receptor (tfR) is stained red, and phalloidin staining of f-actin is shown in yellow. Scale bar 5 µm. (C) *Hp* form smaller microcolonies on the apical cell surface when transferrin receptor expression is knocked down. 3D confocal images of WT colonizing the polarized epithelium 2 days post-infection, on cells either transfected with siRNAs against canine transferrin receptor (ctfR) or eGFP as a control. Bacteria are visualized with anti-*Hp* antibodies (green) and transferrin receptor is stained red. Scale bar 10 µm. (D) Quantification of *Hp* microcolony sizes on cells transfected with siRNAs directed against transferrin receptor or eGFP as a control. Data from 0 and 2 days post-infection are shown. Microcolony sizes were determined by fluorescence volume measured from multiple 3D confocal images. Each point on the graph represents a microcolony. p-values were obtained with a Mann-Whitney statistical test. N.S. indicates no statistical significance.

To directly test if the transferrin receptor pathway is important for *Hp* colonization of the polarized epithelium, we silenced expression of the transferrin receptor during infection and asked whether this affects microcolony growth. We designed siRNAs directed against the canine transferrin receptor and selected two that produce very effective knockdown of transferrin receptor expression (Figure 7A). Cells transfected with a mixture of these two siRNAs were seeded on Transwell filters and allowed to polarize before infection with WT. A first observation was that *Hp* was able to recruit the minimal amount of transferrin receptor still expressed by the host cells to the sites of bacterial microcolonies (Figure 7B). More importantly, *Hp* formed significantly smaller microcolonies on the apical cell surface of monolayers where transferrin receptor expression had been knocked down, as compared to monolayers transfected with a control siRNA (eGFP) (Figures 7C and 7D).

We made use of the fact that the siRNAs directed against the canine transferrin receptor are highly specific and do not cross-react with human transferrin receptor (Figure S8A) to test that the phenotype observed was not due to off-target effects of the siRNAs. In MDCK cells stably expressing human transferrin receptor, knockdown of endogenous canine transferrin receptor expression left expression of human transferrin receptor intact (Figure S8A). *Hp* allowed to colonize the apical surface of these cells formed microcolonies similar in size to those formed by *Hp* colonizing cells transfected with a control siRNA (Figure S8B). This indicates that the decreased ability of *Hp* to colonize the apical cell surface after knockdown of transferrin receptor expression in MDCK cells is specifically due to decreased expression of transferrin receptor in those cells.

Collectively, these results show that host cell transferrin receptor is functionally important in enabling *Hp* colonization of the apical surface of a polarized epithelium. They also suggest that CagA and VacA-mediated perturbation of transferrin/transferrin receptor recycling allows *Hp* to acquire iron from the host cells.

### *Hp* colonization of the polarized epithelium leads to increased apical release of transferrin

**Figure 8:**
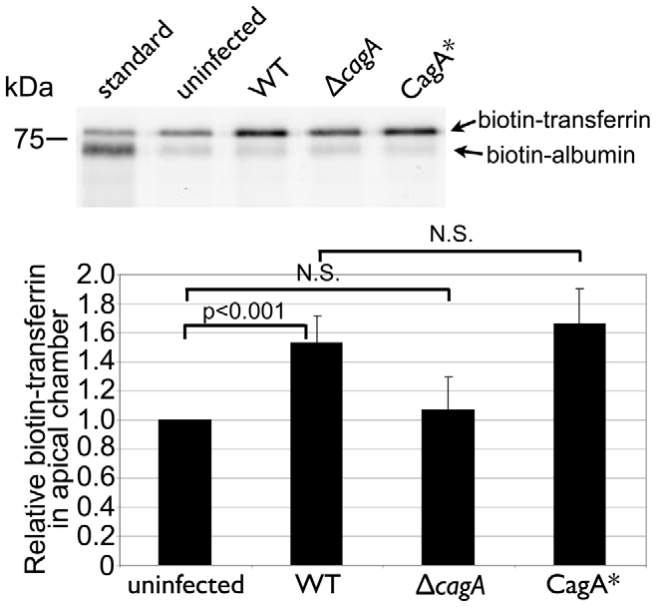
*Hp* colonization of the cell surface leads to transferrin transcytosis. 30 µg of biotin-albumin and 75 µg of biotin-transferrin were added to the basal chamber of uninfected or 2 day-infected polarized monolayers. The apical supernatant was sampled after a 24 hour incubation, and 10 µl of these samples separated by SDS-PAGE, blotted onto nitrocellulose, and the biotinylated albumin and transferrin were visualized with fluorescent streptavidin. The lane labeled “standard” is a 1∶250 dilution of the basal media containing biotin-albumin + biotin-transferrin. CagA* is the complemented Δ*cagA* mutant. Each band was quantified with the LI-COR Odyssey Scanner. Biotin-albumin amounts were used to normalize for loading. The graph depicts the average result from 6 experiments. p-values were obtained with a Wilcoxon signed rank test, using a hypothetical median of 1. N.S. indicates no statistical significance.

Our data suggest a model in which *Hp* colonization of the apical cell surface leads to mis-sorting of a subset of the transferrin/transferrin receptor complex and transcytosis of the complex from the basolateral to the apical surface at the sites of bacterial microcolony growth. We determined whether transferrin is transcytosed apically by adding biotinylated transferrin to the basolateral media and then assaying for the presence of biotinylated transferrin in the apical chamber after a 24-hour incubation period. We detected a 1.5-fold increase in the amount of biotinylated transferrin in the apical chamber of WT-infected monolayers as compared to uninfected monolayers (Figure 8 and Figure S9). This increase was dependent on CagA, since monolayers infected with Δ*cagA* had similar amounts of biotinylated transferrin in the apical chamber as uninfected monolayers (Figure 8). To determine that this increase was due to transcytosis, and to control for possible paracellular leakage of macromolecules, we also added biotinylated albumin to the basolateral chamber. In contrast to transferrin, the small amount of biotinylated albumin detected in the apical chamber was the same irrespective of whether the monolayers were infected with WT or Δ*cagA*, or left uninfected (Figure 8 and Figure S9). Infection with the complemented Δ*cagA* (CagA*) resulted in restoration of the phenotype of increased transferrin transcytosis (Figure 8).

These findings indicate that *Hp* colonization of the apical cell surface results in transcytosis of transferrin to the apical side of the cell.

### CagA confers an adaptive advantage in colonization of the stomach under iron-deplete conditions

*Hp* colonizes multiple niches in the stomach (i.e. free-swimming in the mucus layer vs. the cell surface), and since CagA-negative strains are common in nature, it is likely that *in vivo*, *Hp* utilizes multiple modes of iron acquisition. However, our findings suggest that CagA may be more important for the bacteria when colonizing hosts that are iron-depleted or during conditions of poor dietary iron content. To address this question, we utilized the Mongolian gerbil model of *Hp* infection to determine if the iron status of the host would affect WT or Δ*cagA* colonization.

**Figure 9:**
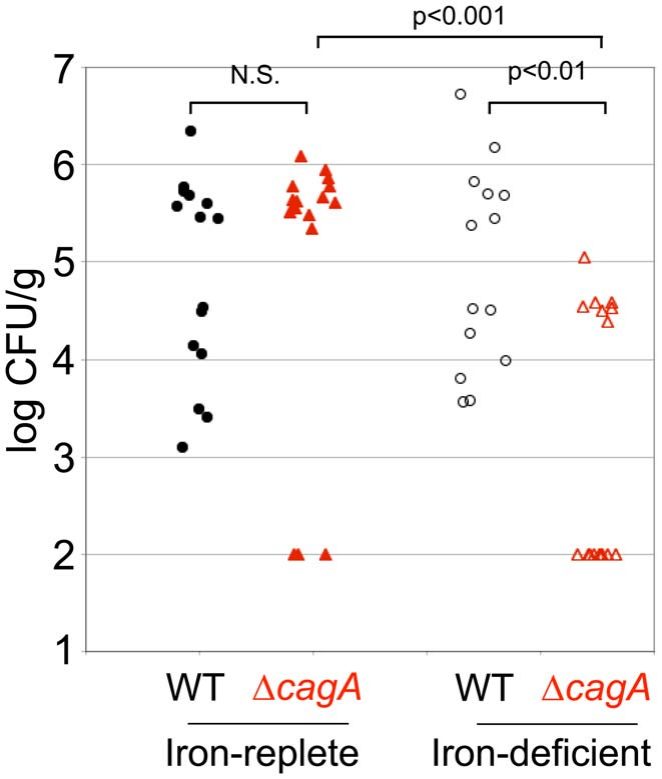
Host iron depletion decreases Δ*cagA* fitness *in vivo*. Mongolian gerbils maintained either on a regular, iron-replete diet, or on an iron-deficient diet were infected with *Hp* strain 7.13 WT or its isogenic Δ*cagA* mutant. 6–8 weeks post-inoculation, the animals were sacrificed and bacterial counts obtained from the stomach. Each point on the graph represents one animal. Animals from which no bacteria could be recovered are represented at 100 CFU/g, which is the limit of detection. p-values were obtained with a Mann-Whitney statistical test. N.S. indicates no statistical significance.

Mongolian gerbils were maintained either on a regular diet containing 250 ppm iron, or an iron-deficient diet containing <1 ppm iron, for 3 weeks prior to infection, and then through the duration of the infection (see Materials and Methods for details). Dietary restriction of iron has been shown to result in decreased host iron levels in mice [42]–[44], and we verified that the animals placed on the iron-deficient diet had reduced iron levels, as measured by inductively coupled plasma-mass spectrometry of liver samples (Figure S10A). For these infections, we used the *Hp* strain 7.13, which has been previously shown to reproducibly colonize the Mongolian gerbil stomach, is able to deliver CagA into host cells, and whose isogenic Δ*cagA* mutant exhibits a defect in colonization of the polarized epithelium *in vitro*, similar to the *Hp* strain G27-MA [8], [45]. In conditions of growth in nutrient-rich broth, 7.13 WT and Δ*cagA* grow with similar kinetics (Figure S10B). We infected Mongolian gerbils with either 7.13 WT or Δ*cagA*, and examined bacterial loads at 6 or 8 weeks after infection. 7.13 WT colonized iron-replete and iron-deficient animals to similar levels (Figure 9). The isogenic Δ*cagA* mutant also colonized iron-replete animals to a similar level as WT. However, 7.13 Δ*cagA* showed a significant decrease in colonization levels in the stomachs of iron-deficient animals, both in comparison to WT in iron-deficient animals and in comparison to Δ*cagA* in iron-replete animals (Figure 9). We also found that bacteria could not be recovered from 9/16 animals in the Δ*cagA*-infected iron-deficient group of animals, unlike the WT-infected iron-deficient group, in which *Hp* could be recovered from all 14 animals infected, at the end of the experiment (Figure 9).

These results mirror our *in vitro* findings that CagA plays an important role in enabling effective acquisition of iron from the host during *Hp* colonization of its gastric epithelial niche.

## Discussion

Bacterial virulence factors are defined as molecules associated with disease. The major virulence factors of *Hp*, CagA and VacA, are epidemiologically linked to disease and possess multiple biological properties that can be deleterious to host cells [46]–[49]. However, understanding how these molecules function during an infection requires asking not just how virulence factors disrupt the host cell, but also how such effects benefit the bacteria. Our previous study had established that *Hp* can grow as microcolonies attached to the apical cell surface of a polarized epithelium, even in conditions where the free-swimming bacteria are rapidly killed [8]. CagA helps the bacteria form microcolonies and exploit this niche by perturbing cell polarity [8]. Here, we extend the concept that bacteria perturb host cell polarity to use the apical cell surface as a replicative niche. We show that bacterial virulence factors alter polarized host intracellular trafficking, suggesting a novel mechanism by which the bacteria are able to acquire essential micronutrients from host cells and colonize the apical cell surface.

We found that exogenous iron added to the media bathing bacterial microcolonies on the apical cell surface partially rescues the *Hp* Δ*cagA* mutant growth defect. This suggests that one of the factors *Hp* extract from epithelial cells is iron, and that one of CagA's benefits to the bacteria involves facilitating iron acquisition from the epithelium. Furthermore, *Hp* is able to colonize the apical membrane of the epithelium even in the presence of excess iron chelators bathing the interstitial side of the epithelium, suggesting that it can acquire iron directly from the host cells without gross disruption of epithelial integrity. We note that attachment of *Hp* to non-polarized host cells had previously been reported to result in upregulation of expression of several annotated iron uptake proteins [50]. Because uptake of iron into host epithelial cells is a polarized process, occurring largely basolaterally through the transferrin/transferrin receptor recycling pathway [22], we probed whether this pathway is manipulated by *Hp* during colonization of the cell surface.

In the presence of an intact epithelial barrier, microbes on the apical surface do not have access to interstitial iron-bound transferrin or its recycling. In addition, partially saturated transferrin, which is the form found in the interstitium, is toxic to *Hp* [21]. However, *Hp* is able to utilize iron from holotransferrin [21], which is the form that eukaryotic cells preferentially bind and uptake due to its higher affinity for the transferrin receptor, as compared to partially saturated or iron-free transferrin [22], [51]. This suggested that *Hp* may be able to utilize the epithelium both as a barrier against the toxic effects of partially saturated transferrin, and as a source of holotransferrin. We observed that the apical cell surface colonization defect of Δ*cagA* mutants can be partially rescued by addition of holotransferrin to the apical chamber, suggesting that intracellular holotransferrin could be one possible iron source for colonizing bacteria.

For *Hp* microcolonies to utilize holotransferrin as an iron source without destroying the epithelium, polarized uptake and recycling of transferrin would have to be perturbed. Both CagA and VacA have biological properties that could be involved in this process. For example, CagA is known to be able to induce receptor tyrosine kinase (RTK)-like signaling [29]–[31], and growth factor RTK activation has been shown to increase uptake of transferrin in other models [33]. CagA has also been shown to affect cell polarity and the assembly of the epithelial junctions [35]–[37], [45], both of which could influence sorting of basolateral molecules [52]–[54]. VacA, another major virulence factor of *Hp*, has previously been reported to affect endosomal trafficking [38]. We therefore hypothesized that CagA and VacA could have effects on the host cell transferrin/transferrin receptor recycling pathway.

We found that CagA injected into host cells by *Hp* growing as microcolonies on the apical cell surface increased internalized transferrin. This required signaling via the EPIYA motifs in the C-terminus of the protein, which are important in the activation of RTK-like signaling [29], [30], [32]. We also observed that a subset of the transferrin receptor population is mislocalized from the basolateral surface specifically to sites of bacterial microcolonies on the apical cell surface. In our model system, this mislocalization is largely dependent on the action of VacA, as a Δ*vacA Hp* mutant failed to recruit transferrin receptor apically. We were able to show a direct involvement of the transferrin/transferrin receptor recycling process in the colonization of the polarized epithelium by *Hp*, since siRNA knockdown of transferrin receptor expression resulted in decreased growth of *Hp* microcolonies on the cell surface. Our model suggests that *Hp* colonizing the apical cell surface induce mis-sorting of a subset of the transferrin/transferrin receptor complex apically. In accord with this, we observed significantly increased transcytosis of transferrin from the basal to the apical compartment and its release into the apical media when the epithelium is infected by WT *Hp*.

Several important questions remain regarding this model. First, it is clear that iron acquisition from the epithelium is only one of several mechanisms of iron acquisition by *Hp*. *Hp* can live as a free-swimming population *in vitro* without the need for contact with epithelial cells. *In vivo*, *Hp* may obtain iron through several mechanisms and from various sources. For example, *Hp* residing as free-swimming bacteria in the mucus layer may obtain iron conjugated in mucus glycoproteins, or perhaps from the release of dietary iron by the acidic stomach lumen, or from inflammatory exudates, although these potential sources need study. Of note is that in addition to its role in causing peptic ulcers and its association with gastric cancer, *Hp* infection has recently been associated with iron deficiency anemia unrelated to blood loss [3]. Indeed, the latest Maastricht Consensus Report recommends diagnosis and treatment of *Hp* in cases of unexplained iron deficiency anemia [55]. There are now several reports in the literature showing that *Hp* eradication improved or cured previously unexplained cases of iron deficiency anemia [4], [56], [57]. Second, it will also be important in future studies to directly visualize the transfer of iron from the host to the bacteria and the precise molecular mechanisms involved. The observation that specific basolateral molecules, such as the transferrin receptor, are highly concentrated at sites of bacterial microcolonies in comparison to the rest of the apical membrane suggests that these sites are a very specialized and enriched microenvironment. However, methods that allow the visualization and quantification of iron in a spatially defined manner at sub-micrometer resolution are still at early stages of development [58], [59]. Technical limitations such as these, as well as difficulties associated with maintaining a polarized monolayer in which the transferrin receptor or other important host molecules have been downregulated over prolonged periods of time, will first have to be overcome before the exact mechanisms by which *Hp* extracts iron from the host epithelium can be fully understood. Several of the known effects of CagA and VacA on host cell physiology may have important roles in altering trafficking of transferrin and other molecules for the benefit of colonizing bacteria. For example, we showed here that signaling through the EPIYA motifs of CagA increase host internalization of transferrin. CagA also has multiple effects mimicking growth factor activity which could influence the way in which gastric epithelial cells uptake and process nutrients that could be used by colonizing *Hp* [29]–[31], [60]. In addition, we had previously reported that CagA's perturbation of host cell polarity via its actions on Par1b are important in enabling *Hp* colonization of the apical cell surface [8]. Of note, one of Par1b's functions is in organizing microtubules, and it has been speculated to play a crucial role in regulation of intracellular vesicle transport [61]. In addition to its effects on endosomal trafficking, VacA has been shown to act as a pore on membranes, and may independently increase transcellular and paracellular flow of iron and other essential factors [48], [62], [63]. We did not observe transferrin receptor mislocalization to sites of bacterial attachment in preliminary experiments with purified VacA added exogenously to Δ*vacA*-infected polarized cell monolayers (data not shown). The delivery of VacA can be local through contact by adhered bacteria [64], as well as at a distance through diffusion of soluble VacA [48]. Since localized VacA delivery during cellular infection may not be equivalent to generalized intoxication, it will be interesting to define how these two forms of delivery differ in their effects on polarized epithelia. It is also possible that VacA is necessary, but not sufficient, for this process.

The potential cooperation between CagA and VacA to affect polar transport of micronutrients to colonizing microcolonies is exciting, since CagA and certain VacA alleles have been linked genetically [65], and possible interplay between their functions have been reported [66]–[70]. Different subtypes of CagA and VacA may also have varied activities in aiding *Hp* colonization of the epithelium. For example, the “East Asian” form of CagA has been reported to bind more strongly to Par1b than the “Western” form of CagA [71]. How do such differences translate to the ability of different strains of the bacteria to colonize the epithelium? Our findings suggest that CagA and VacA may act in concert in a novel way of inducing the host epithelium to relinquish essential micronutrients that does not necessitate destroying the epithelium or causing gross leakage of interstitial macromolecules into the lumen. Further studies will reveal the exact pathways that are usurped by CagA and VacA in altering host transferrin trafficking, visualize how iron is delivered to *Hp* on the cell surface, and perhaps uncover additional iron acquisition methods by *Hp*.

Our use of a simplified experimental system allowed us to focus on the microenvironment of the apical cell surface as a replicative niche, and to uncover one possible mechanism by which *Hp* may obtain iron from host epithelial cells. We subsequently found that Δ*cagA* mutant *Hp* have a decreased ability to colonize the stomachs of Mongolian gerbils that are iron-deficient whereas WT bacteria do not, indicating that CagA aids *Hp* in acquiring iron from the host during *in vivo* bacterial colonization. Our findings also raise the question of how host micronutrient levels impact the pathogenesis of colonizing *Hp*. For example, might decreased levels of micronutrients in the host lead to increased pathogenicity of the bacteria? We note here that expression of both *cagA* and *vacA* have been reported to be upregulated in conditions of iron starvation *in vitro* [9], [72], and that the ferric uptake regulator (Fur) protein indirectly regulates *vacA* expression [72], [73]. Previous epidemiological data had suggested an association between iron deficiency and gastric cancer, although this data predates our understanding of the role of *Hp* infection [74]. It will now be interesting to examine epidemiologically the possible roles of CagA and VacA in the context of iron deficiency in human populations.

We suggest that manipulation of host epithelial polarity is akin to utilizing the epithelium as a filter to acquire essential micronutrients while maintaining a barrier that protects the microbes from innate immune defenses present in the interstitial space. Perturbation of host epithelial polarity by *Hp* appears to be a specific and subtle process, not due to a generalized loss of epithelial polarity, since biotinylated proteins from the basolateral membrane form patches specifically under apical bacterial microcolonies, and not all basolateral proteins are found in these membrane patches. Although we focused on transferrin receptor mislocalization in this study, the technique of basal biotinylation of host membrane proteins in infected monolayers suggests that there are multiple proteins that are mislocalized to the apical sites of bacterial growth. The identity of these proteins may shed other important insights into the way microbes colonize the epithelial surface. CagA and VacA play major roles in this mislocalization, but other bacterial factors do appear to be involved as well. The ability to manage host epithelial polarity is emerging as an important facet of bacterial-host interactions. For example, *Listeria monocytogenes* has been shown to take advantage of cell polarity changes for invasion [41], [75], and mislocalization of basolateral proteins to the apical cell surface as a result of bacterial infection has also been described for other pathogens such as enteropathogenic *Escherichia coli* and *Pseudomonas aeruginosa* [76], [77]. What are the mechanisms by which specific basolateral molecules are sequestered to the sites of bacterial microcolonies? During *P. aeruginosa* infection of polarized epithelia, phosphatidylinositol 3, 4, 5-trisphosphate (PIP3), a basolateral membrane lipid important in cellular signaling and polarity, is mislocalized to the sites of bacterial attachment on the apical cell surface [77], [78]. PIP3 is an intriguing candidate in the context of our results with the transferrin receptor, as it has recently been shown to also localize to recycling endosomes, and to be important for the proper sorting of recycling cargo, such as the transferrin receptor [79]. Furthermore, both CagA and VacA have been implicated in the activation of the phosphatidylinositol 3-kinase pathway, which catalyzes production of PIP3 [80], [81]. It will be interesting to test if PIP3 is recruited to sites of *Hp* microcolonies on the apical cell surface, and whether PIP3 function is involved in *Hp*'s colonization of the polarized epithelium.

In summary, our results show that iron is one important factor that *Hp* is able to obtain from host cells during colonization of the apical cell surface, and illustrate a way in which CagA and VacA work in concert to aid the bacteria in establishing a replicative niche. We hypothesize that growth on the epithelial surface involves more than just iron acquisition from the host. For example, it has previously been shown that *Hp* can acquire cholesterol directly from host cells via contact [82]. We speculate that *Hp* has evolved sophisticated mechanisms to manipulate host cell physiology for its own benefit, and that these features have side effects that result in disease in a subset of the infected population. Instead of destroying the epithelium as may occur in some types of acute bacterial infection, mucosal colonizers like *Hp* use more subtle mechanisms of local epithelial perturbation. We propose that mucosal colonizers may be utilizing the polarized epithelium as a “filter” both to protect themselves from potentially toxic host defense molecules, and to selectively extract micronutrients that are present inside the host in a form that is usable by the bacteria. Future exploration of the nature of host proteins associated with apical bacterial microcolonies, and the possible role of other bacterial factors in perturbing polarity, will give us a better understanding of how *Hp* and other mucosal colonizers affect the epithelial surface for their benefit.

## Materials and Methods

### Ethics statement

All animal experiments were performed in accordance to NIH guidelines, the Animal Welfare Act, and US federal law. Such experiments were carried out with the approval of the Institutional Animal Care and Use Committee of Vanderbilt University, which has been accredited by the Association of Assessment and Accreditation of Laboratory Animal Care International (AAALAC). All animals were housed in an AAALAC-accredited research animal facility fully staffed with trained technical, husbandry, and veterinary personnel.

### Cell culture

Madin-Darby Canine Kidney II (MDCK) cells (kindly provided by W. James Nelson, Stanford University, Stanford, CA) [83], and MDCK cells stably expressing human transferrin receptor (kindly provided by Suhaila White and Suzanne Simon, The Salk Institute, La Jolla, CA) [25], were maintained in DMEM (Gibco) containing 5% fetal bovine serum (FBS) (Gibco), at 37°C in a 5% CO_2_ atmosphere. Caco-2 cells (ATCC) were maintained in DMEM containing 10% FBS, at 37°C in a 5% CO_2_ atmosphere. Polarized MDCK and Caco-2 monolayers were cultured by seeding cells at confluent density onto 12 mm, 0.4 µm-pore polycarbonate tissue culture inserts (Transwell filters; Corning Costar). Polarized MDCK monolayers were maintained as previously described [8]. Caco-2 cells on Transwell filters were allowed to fully polarize for 3 weeks before use in assays. Apical medium was changed to DMEM one day after seeding, and basal medium (DMEM + 10% FBS) was changed daily during this time.

### *Hp* strains and culture

*Hp* strain G27-MA and its isogenic Δ*cagA* mutant have been previously described [8], [35]. Complementation of G27-MA Δ*cagA* has been previously described [8]. An isogenic Δ*vacA* mutant of strain G27-MA was constructed by deletion of the *vacA* open reading frame (ORF) beginning from the start codon to 14 base pairs before the end of the ORF, and replacement with the *aphA* gene (conferring kanamycin resistance), by a PCR based method without recombinant cloning [84], [85]. Complementation of G27-MA Δ*vacA* was accomplished by natural transformation with a construct containing the *vacA* ORF with the *cat* gene (conferring chloramphenicol resistance) immediately downstream, flanked by the upstream and downstream regions of *vacA* to allow for homologous recombination. Verification of the Δ*vacA* mutant and of the complemented strain (VacA*) was performed by immunoblotting bacterial whole cell lysates with a polyclonal rabbit anti-VacA antibody (Austral Biologicals) (Figure S11A). Immunoblots of bacterial whole cell lysates with a rabbit anti-CagA-N-terminus antibody [8] or a rabbit anti-VacA antibody did not show significant differences in CagA or VacA expression resulting from deletion of *vacA* or *cagA* respectively (Figure S11B). A G27-MA Δ*cagA*Δ*vacA* double mutant was obtained by natural transformation of the single chloramphenicol-resistant Δ*cagA* mutant with the Δ*vacA* deletion construct, and selection on Columbia blood agar plates containing 25 µg/ml kanamycin and 25 µg/ml chloramphenicol. G27-MA carrying a mutated CagA that cannot be phosphorylated (EPISA) was constructed by transformation with a previously described allele [35], with the *aphA* gene inserted immediately downstream of the mutant CagA sequence for selection of transformants. *Hp* strain 7.13 and its isogenic Δ*cagA* mutant have been previously described [8], [45]. Routine culture of *Hp* on Columbia blood agar plates and co-culture of *Hp* with MDCK cells were as previously described [35], [86]. Unless otherwise indicated, *Hp* from co-cultures were used for infections. Co-culture media for *Hp* with MDCK cells consists of DMEM + 5% FBS +10% Brucella broth +10 µg/ml vancomycin.

### Generation of holotransferrin

Transferrin was saturated with iron essentially as previously described [87]. 9 mg of human transferrin (Sigma) was added to 1.5 ml of 0.25 M Tris-Cl, pH 8, containing 10 µM of NaHCO_3_. 30 µl of a mixture of 100 mM disodium nitrilotriacetate (Sigma) and 12.5 mM FeCl_3_ (Sigma) was then added to the solution. After incubation at 37°C for 1 hour, the sample was passed through a HiTrap desalting (Sephadex G-25 Superfine) column (GE Healthcare), previously equilibrated with a solution of 0.02 M Tris-Cl, pH 7.4, containing 0.15 M NaCl. The ratio of absorbance at 465 nm to 280 nm was measured to provide an estimate of the amount of iron bound by transferrin [88].

To remove unbound iron from the preparations, the samples were subsequently treated with Chelex resin (BioRad) [21], according to the manufacturer's recommended batch method protocol.

### *Hp* growth assays in broth

For *Hp* growth assays in media without cells, *Hp* grown overnight on Columbia blood agar plates were resuspended in DMEM, and aliquots inoculated into the appropriate media in 6-well plates. Where added, ferric chloride (FeCl_3_, Sigma) was added at a final concentration of 100 µM, and human transferrin (Sigma) was added at a final concentration of 75 µg/ml. Data are shown as means ± SD. We also tested holotransferrin (prepared as described above) added to DMEM at a final concentration of 75 µg/ml, which does not allow growth of *Hp* without the presence of cells (Figure S1C).

### *Hp* growth assays on polarized monolayers

Infection of polarized MDCK monolayers with *Hp* was carried as previously described [8]. In brief, *Hp* (∼10^8^ bacteria/ml) were added to the apical chamber, allowed to adhere for 5 minutes, and cell monolayers washed 5 times with fresh DMEM to remove non-adherent bacteria. Appropriate media was added back to the apical chamber, and the cells incubated at 37°C in a 5% CO_2_ atmosphere. Basal media was changed daily. After sampling for colony forming unit (CFU) counts from the apical chamber each day, cell monolayers were washed 3 times with fresh DMEM, before appropriate media added back and the cells returned to the incubator. Data are shown as means ± SD. *Hp* express both CagA and VacA during colonization of the polarized epithelium as evaluated by immunoblotting (Figure S12A). We had showed previously that *Hp* is able to deliver CagA into MDCK cells [8], and verified here that *Hp* growing on the apical cell surface is also able to deliver VacA into the host cells by immunofluorescence staining with a mouse monoclonal anti-VacA antibody (Santa Cruz Biotechnology) (Figure S12B).

DMEM is iron-poor, containing 0.248 µM ferric nitrate (Invitrogen media formulation), in contrast to Brucella broth media often used in *Hp* culture, which has been reported to contain 13.9 µM of iron [89]. Where used, FeCl_3_ (Sigma) was added to the media in the apical chamber at a final concentration of 100 µM (unless otherwise stated). Holotransferrin (prepared as described above) or transferrin (Sigma) was added to the media in the apical chamber at 75 µg/ml when used. For experiments with transferrin added to the co-culture media in the basal chamber, transferrin was added at a final concentration of 75 µg/ml.

### Confocal immunofluorescence microscopy and antibodies

Samples were processed for confocal immunofluorescence as previously described [41]. Mouse anti-ZO-1 and mouse anti-transferrin receptor antibodies (Zymed) were used at 1∶300 dilution. Mouse monoclonal antibody rr1, which recognizes an extracellular epitope of E-cadherin [41], [90], [91], was used at 1∶100 dilution. Chicken anti-*Hp* antibodies [35] were used at 1∶200 dilution. Mouse anti-VacA (Santa Cruz Biotechnology) was used at 1∶100 dilution. Anti-IgG Alexa-fluor conjugated antibodies of appropriate fluorescence and species reactivity (Molecular Probes) were used for secondary detection. For transferrin and transferrin receptor experiments, an anti-chicken IgG Dylight 405 conjugated antibody (Rockland Immunochemicals) was used for secondary detection of the chicken anti-*Hp* antibodies. We did not use the 488 nm channel in these experiments, and visualized transferrin or transferrin receptor in the 594 nm channel to avoid overlap of the fluorescence spectra and prevent signal bleed-through from one channel into the next. Alexa-fluor 647-conjugated phalloidin (Molecular Probes) was pseudocolored yellow in these experiments. For all other experiments, either Alexa-fluor 594 or 647-conjugated phalloidin were used for visualization of the actin cytoskeleton and pseudocolored red or blue respectively. Nuclei were visualized with DAPI (Invitrogen). Samples were imaged with a BioRad MRC-1024 confocal microscope, or with a Zeiss LSM 700 confocal microscope, and z-stacks reconstructed into 3D using Volocity software (Improvision). Quantification of microcolony sizes was carried out as previously described [8]. Statistical differences between the data sets were determined by non-parametric Mann-Whitney test.

### Fluorescent transferrin uptake assays

MDCK cells stably expressing human transferrin receptor were seeded on Transwell filters and allowed to polarize before use in assays. Polarized cells were left uninfected or infected with *Hp* from the apical side for two days. For assays with polarized Caco-2 cells, *Hp* were infected from the apical side for 18 hours. Monolayers were washed 3 times apically and 5 times basally with fresh, pre-warmed DMEM. DMEM was added back to the apical chamber, and DMEM + 25 µg/ml human transferrin conjugated to Alexa Fluor 594 (Invitrogen) added to the basal chamber. The samples were then incubated on ice for 30 minutes. After this time, monolayers were washed 5 times with fresh DMEM basally. Samples were then either immediately fixed and processed for confocal immunofluorescence, or co-culture media added back to the basal chamber and the samples incubated for 30 minutes at 37°C in a 5% CO_2_ atmosphere before fixation and processing.

For quantification of transferrin signal, we randomly sampled 300 µm X 300 µm optical fields by confocal microscopy. Stacks containing the full thickness of the monolayers were acquired at 0.5 µm z-steps. The stacks were reconstructed in 3D and the fluorescence sum of the transferrin signal present in the monolayers was measured. The background fluorescence was calculated from voxels imaged below the monolayers in each sample. Statistical differences between the data sets were determined by non-parametric Mann-Whitney test.

### Western blots

Lysates were prepared for Western blots as previously described [8]. For samples from polarized monolayers on Transwell filters, cells from 3 filters were pooled for each lysate. Samples were separated by SDS-PAGE, and transferred to nitrocellulose membranes for immunoblotting. Mouse anti-transferrin receptor (Zymed) was used at 1∶5000. Mouse anti-GAPDH (EMD Chemicals Inc.) was used at 1∶10000. Rabbit anti-CagA-N-terminus [8] was used at 1∶10000. Rabbit anti-VacA (Austral Biologicals) was used at 1∶2500. Goat anti-mouse or anti-rabbit IgG Alexa-fluor 660-conjugated antibodies (Molecular Probes) were used for secondary detection. To visualize total protein, SDS-PAGE gels were stained with Coomassie Blue (Sigma). A LI-COR Odyssey Scanner was used for signal detection (LI-COR Biosciences).

### Assay for mislocalizaton of transferrin receptor to the apical surface of polarized cells

Polarized monolayer samples were fixed and non-permeabilized, apical staining carried out as previously described [41]. For quantification of transferrin receptor signal associated with bacterial microcolonies, 100 µm X 100 µm optical fields were randomly sampled by confocal microscopy. The 3D reconstructions of the confocal stacks were used to collect the fluorescence voxel volume of each microcolony stained with anti-*Hp* antibodies, and the fluorescence sum of the transferrin receptor signal associated with the microcolonies. For background measurements, regions on the apical cell surface of ∼20 µm^3^ with no bacteria were also measured for the fluorescence sum of the transferrin receptor signal present. Statistical differences between the data sets were determined by non-parametric Mann-Whitney test.

### Selective cell surface biotinylation

Polarized monolayers were rinsed 3 times with Ringer's buffer (154 mM NaCl, 7.2 mM KCl, 1.8 mM CaCl_2_, 10 mM HEPES, pH 7.4). Ringer's buffer containing 200 µg/ml sulfo-NHS-SS-biotin (Pierce) was added to either the basal or apical chambers for selective basal vs. apical membrane protein biotinylation [40]. Samples were incubated on ice for 30 minutes. The cells were then washed 5 times with Tris-saline (120 mM NaCl, 10 mM Tris-HCl, pH 7.4), and 3 times with DMEM. Samples were then either immediately fixed and processed for confocal immunofluorescence, or incubated for 30 minutes at 37°C in a 5% CO_2_ atmosphere before fixation and processing. Non-permeabilized, apical staining with Alexa Fluor 488-conjugated streptavidin (Molecular Probes) was carried out as described previously [41]. Quantification of biotin signal associated with bacterial microcolonies was carried out as described for the transferrin receptor. Statistical differences between the data sets were determined by non-parametric Mann-Whitney test.

### Gene expression knockdowns with siRNA

siRNAs directed against canine transferrin receptor were designed by Ambion, Applied Biosystems Inc., using their *Silencer* Select siRNA design algorithm. Two canine transferrin receptor-targeted siRNAs were used in the experiments here – 5′ GCAGAAAAGUUGUUUGAAA, and 5′ CCUAUGAUCUUGAAUUGAA. A *Silencer* Select Validated siRNA directed against human transferrin receptor (5′ GGUCAUCAGGAUUGCCUAA) and a control enhanced green fluorescent protein (eGFP) *Silencer* siRNA (#AM4626) were also obtained from Ambion. siRNAs were transfected into MDCK cells using a reverse transfection protocol with Lipofectamine RNAiMAX Transfection Reagent (Invitrogen). For each well to be transfected in a 6-well plate, 50 pmoles of RNAi duplex and 7.5 µl of Lipofectamine RNAiMAX Transfection Reagent were gently mixed in 500 µl of OPTI-MEM I Reduced Serum Medium. The mixture was incubated at room temperature for 20 minutes, and 5×10^5^ cells suspended in 2 ml of DMEM + 5% FBS added to the mixture. The cells were then incubated at 37°C in a 5% CO_2_ atmosphere for 1–3 days. Samples were collected for Western blotting as previously described [8].

For *Hp* infection of polarized cells transfected with siRNA, cells reverse-transfected with siRNA as above were trypsinized after a 24 hour incubation at 37°C in a 5% CO_2_ atmosphere, and seeded at high confluent density onto Transwell filters. 30 hours after seeding, infection of the polarized epithelial cells was then carried out as described earlier for the Transwell *Hp* growth assays, with DMEM present in both the apical and basal chambers. Quantification of microcolony sizes was carried out as previously described [8]. Statistical differences between the data sets were determined by non-parametric Mann-Whitney test.

### Transferrin transcytosis assay

MDCK cells stably expressing human transferrin receptor were seeded on Transwell filters and allowed to polarize before use in assays. Co-culture media was added basally, and DMEM apically. Polarized cells were left uninfected or infected with *Hp* from the apical side for 2 days. After this time, the media in the Transwell basal chamber was replaced with co-culture media containing 50 µg/ml biotinylated transferrin (Invitrogen) and 20 µg/ml biotinylated albumin (Sigma). The basal chamber contains 1.5 ml of media, while the apical chamber contains 0.5 ml of media. After a 24 hour incubation at 37°C in a 5% CO_2_ atmosphere, the media from the apical chamber was collected and diluted 1∶1 in 2X SDS-sample buffer. Samples were separated on a SDS-PAGE gel, and transferred to nitrocellulose membranes for immunoblotting. Detection of biotinylated transferrin and biotinylated albumin was carried out by blotting with streptavidin conjugated to Alexa Fluor 660 (Molecular Probes), and scanning on a LI-COR Odyssey Scanner (LI-COR Biosciences). The detection limit and linear range of measurements of the biotinylated transferrin and biotinylated albumin were determined from standard curves generated by use of a dilution series of the co-culture media containing 50 µg/ml biotinylated transferrin and 20 µg/ml biotinylated albumin, diluted 1∶1 in SDS-sample buffer.

### Experimental animal infections

Mongolian gerbils (Harlan Laboratories) were placed on a regular, iron-replete diet (Modified TestDiet AIN-93M with 250 ppm iron), or an iron-deficient diet (Modified TestDiet AIN-93M with no iron) (TestDiet, Purina Mills, LLC), for 3 weeks prior to infection. Animals were then inoculated with either *Hp* strain 7.13 WT or a 7.13 Δ*cagA* mutant, and sacrificed 6–8 weeks post-inoculation [92], [93]. The iron-replete and iron-deficient diets were maintained as appropriate for each group of animals throughout the course of the experiment. Colonization was determined by quantitative culture [92], [93], and liver samples were also collected at the time of sacrifice for analysis of total iron content. Iron analysis was performed using inductively coupled plasma-mass spectrometry, carried out by Applied Speciation and Consulting, LLC. Statistical differences between the data sets were determined by non-parametric Mann-Whitney test.

## Supporting Information

Figure S1 Addition of iron to DMEM is not sufficient to support *Hp* growth in the absence of host cells. (A) Addition of iron to DMEM is not sufficient to support *Hp* growth in liquid culture. Plate-grown WT was used to inoculate co-culture media (solid black line), DMEM (red line) or DMEM containing 100 µM ferric chloride (Fe^3+^; dashed red line), in the absence of host cells. Samples were taken over time and plated for CFU counts. (B) Partially saturated transferrin can inhibit *Hp* growth in broth. Plate-grown WT was used to inoculate co-culture media (solid black line), DMEM (red line) or co-culture media containing 75 µg/ml partially saturated transferrin (Tf; dashed black line), in the absence of host cells. Samples were taken over time and plated for CFU counts. (C) Addition of holotransferrin to DMEM is not sufficient to support *Hp* growth in liquid culture. Plate-grown WT was used to inoculate co-culture media (solid black line), DMEM (red line) or DMEM containing 75 µg/ml holotransferrin (holotf; dashed red line), in the absence of host cells. Samples were taken over time and plated for CFU counts.
(TIF)

Figure S2 Characterization of MDCK cells stably expressing human transferrin receptor. (A) Basolateral localization of transferrin receptor. Fluorescent transferrin (red) was added to the basal chamber of a polarized monolayer of MDCK cells stably expressing human transferrin receptor, and incubated for 30 minutes on ice before fixation. A cross section through the monolayer is shown. Scale bar 10 µm. (B) MDCK cells stably expressing human transferrin receptor were polarized on Transwell filters and infected with WT or Δ*cagA*. Co-culture media (+) was added basally and DMEM (−) added apically. Samples were taken daily from the apical chamber and plated for CFU counts.
(TIF)

Figure S3 CagA-dependent increase of internalized transferrin occurs in multiple polarized epithelial lines. (A) *Hp* colonization affects Caco-2 cell transferrin internalization. Polarized Caco-2 cells were infected for 18 hours with WT or Δ*cagA*. Fluorescent transferrin was added to the basal chamber and incubated on ice for 30 minutes, unbound transferrin washed away, then further incubated for 30 minutes at 37°C to allow uptake of bound transferrin. The graph shows quantitative data of the transferrin fluorescence signal at 30 minutes post-uptake, determined from multiple 3D confocal images. p-value was obtained with a Mann-Whitney statistical test. (B and C) Differences in WT vs. Δ*cagA* effects on host cell transferrin internalization are not due to differences in bacterial numbers. Polarized MDCK cells stably expressing human transferrin receptor in the Transwell system, with 100 µM ferric chloride added to the apical chamber, were infected for 2 days with WT or Δ*cagA*. Fluorescent transferrin uptake assay was carried out as in (A). The transferrin fluorescence signal was quantified at 30 minutes post-uptake (B). p-value was obtained with a Mann-Whitney statistical test. 3D confocal images of the monolayers at 30 minutes post-uptake of fluorescent transferrin (red) are shown in (C). Bacteria are visualized with anti-*Hp* antibodies (green). Scale bar 20 µm.
(TIF)

Figure S4 Mislocalization of transferrin receptor to sites of bacterial microcolonies is specific. (A) Initial attachment of *Hp* to host cells does not result in transferrin receptor mislocalization. Polarized MDCK cells in the Transwell system were infected with WT for 5 minutes and fixed immediately. Apical staining with anti-transferrin receptor antibodies was carried out on non-permeabilized samples. Bacteria are visualized with anti-*Hp* antibodies (blue), transferrin receptor (tfR) is stained red, and phalloidin staining of f-actin is shown in yellow. 3D confocal images are shown. Scale bar 5 µm. (B) Not all basolateral proteins are mislocalized to bacterial microcolonies. Polarized MDCK cells on Transwell filters were infected with WT for 2 days, then fixed and stained with antibodies against E-cadherin (red) either from the apical surface without permeabilization (top panels), or with permeabilization (bottom panels, cross-section). Bacteria are stained with anti-*Hp* antibodies (blue), and phalloidin staining of f-actin is shown in yellow. Scale bars 5 µm.
(TIF)

Figure S5 Apical mislocalization of transferrin receptor to sites of bacterial microcolonies occurs in multiple cell types. Polarized Caco-2 cells in the Transwell system were infected with WT, Δ*cagA*, or Δ*vacA* for 18 hours. Apical staining with anti-transferrin receptor antibodies was carried out on non-permeabilized samples. Bacteria are visualized with anti-*Hp* antibodies (blue), transferrin receptor (tfR) is stained red, and phalloidin staining of f-actin is shown in yellow. 3D confocal images are shown, and cross-sectional view is also presented for WT (second row). Scale bars 5 µm.
(TIF)

Figure S6 CagA and VacA both contribute to mislocalization of basolateral proteins to the apical surface at sites of bacterial attachment. Polarized cells infected apically with WT, Δ*cagA*, Δ*vacA*, or Δ*cagA*Δ*vacA* for 2 days were selectively biotinylated on ice at the basolateral surface, then incubated for 30 minutes at 37°C before fixation and apical streptavidin staining. Each point represents the total fluorescence intensity of apically-exposed biotin associated with a microcolony, divided by the number of bacteria present in that microcolony. p-values were obtained with a Mann-Whitney statistical test.
(TIF)

Figure S7 Δ*cagA*, Δ*vacA*, and Δ*cagA*Δ*vacA* mutants grow as well as WT in the presence of nutrients. Polarized MDCK cells in the Transwell system were infected with WT, Δ*cagA*, Δ*vacA*, or Δ*cagA*Δ*vacA*. Co-culture media (+) was added both apically and basally. Samples were taken daily from the apical chamber and plated for CFU counts.
(TIF)

Figure S8 Effect of canine transferrin receptor knockdown is specific. (A) siRNA against canine transferrin receptor is specific. MDCK cells stably expressing human transferrin receptor were mock transfected, or transfected with siRNA against canine or human transferrin receptor (tfR). After polarization, fluorescent human transferrin (red) was added to the basal chamber, and incubated for 30 minutes on ice before fixation. Cross sections through the monolayers are shown. Nuclei are stained with DAPI (blue), phalloidin staining of f-actin is shown in yellow, and cellular tight junctions are visualized with anti-ZO-1 (green). Scale bar 10 µm. (B) Quantification of *Hp* microcolony sizes on MDCK cells stably expressing human transferrin receptor, transfected with siRNAs directed against canine transferrin receptor (ctfR) or eGFP as a control. Data from 0 and 2 days post-infection are shown. Microcolony sizes were determined from multiple 3D confocal images. Each point on the graph represents a microcolony. p-values were obtained with a Mann-Whitney statistical test. N.S. indicates no statistical significance.
(TIF)

Figure S9 Detection limit and linear range of biotin-albumin and biotin-transferrin measurements. Samples of co-culture media containing biotin-albumin (range 0.4 ng to 10 ng) and biotin-transferrin (range 1 ng to 25 ng) were loaded and separated by SDS-PAGE and transferred to a nitrocellulose membrane. The membrane was probed with Alexa-fluor 647-conjugated streptavidin and bands visualized by the LI-COR Odyssey Scanner and quantified. Arbitrary units were used for the integrated intensity graph. The data was plotted as integrated intensity minus the background (bkg). Best-fit linear curves for the data are shown, as are the linear formulas and their fit.
(TIF)

Figure S10 Iron depletion of Mongolian gerbils and *in vitro* growth curves of *Hp* strain 7.13 WT and its isogenic Δ*cagA* mutant. (A) Dietary iron restriction leads to decreased iron levels. Mongolian gerbils were maintained on a regular, iron-replete diet, or on an iron-deficient diet for 3 weeks prior to WT *Hp* infection, and throughout the course of the 6-week infection. Liver samples were analyzed by inductively coupled plasma-mass spectrometry (Applied Speciation and Consulting, LLC). p-values were obtained with a Mann-Whitney statistical test. (B) *Hp* strain 7.13 WT and its isogenic Δ*cagA* mutant grow equally well in nutrient-rich broth. Growth of *Hp* strain 7.13 WT and Δ*cagA* in Brucella broth + 10% FBS was followed over 30 hours by measurement of optical density at 600 nm (OD_600_). Similar results were obtained in 3 independent experiments.
(TIF)

Figure S11 Verification of mutants by immunoblot. (A) Complementation of Δ*vacA* restores VacA expression. Lysates of *Hp* grown on Columbia blood agar plates were separated by SDS-PAGE, then either stained with Coomassie Blue or transferred to a nitrocellulose membrane and immunoblotted with polyclonal antibodies against VacA. VacA* is the complemented Δ*vacA* mutant. (B) CagA and VacA expression is not affected by deletion of *vacA* and *cagA* respectively. Lysates of *Hp* grown on Columbia blood agar plates were separated by SDS-PAGE, then either stained with Coomassie Blue or transferred to a nitrocellulose membrane and immunoblotted with polyclonal antibodies against CagA or VacA.
(TIF)

Figure S12 CagA and VacA expression by *Hp* during infection in the Transwell system. (A) *Hp* colonizing the polarized epithelium express CagA and VacA. Polarized MDCK cells in the Transwell system were infected with WT. Free-swimming bacteria were washed away with DMEM and lysates of infected cells were collected at 2 or 5 days post-infection, separated by SDS-PAGE, then either stained with Coomassie Blue or transferred to a nitrocellulose membrane and immunoblotted with antibodies against CagA or VacA. (B) *Hp* colonizing the polarized epithelium deliver VacA into the host cells. Polarized MDCK cells in the Transwell system were infected with WT for 2 days, then fixed and stained with antibodies against *Hp* (green) and VacA (red). Phalloidin staining of f-actin is shown in blue. Scale bar 10 µm.
(TIF)
