# Supplementary figures and images for: Helicobacter pylori Perturbs Iron Trafficking in the Epithelium to Grow on the Cell Surface
Source: PLoS Pathog. 2011 May 12;7(5):e1002050. doi: 10.1371/journal.ppat.1002050 (PMC3093365; doi:10.1371/journal.ppat.1002050)

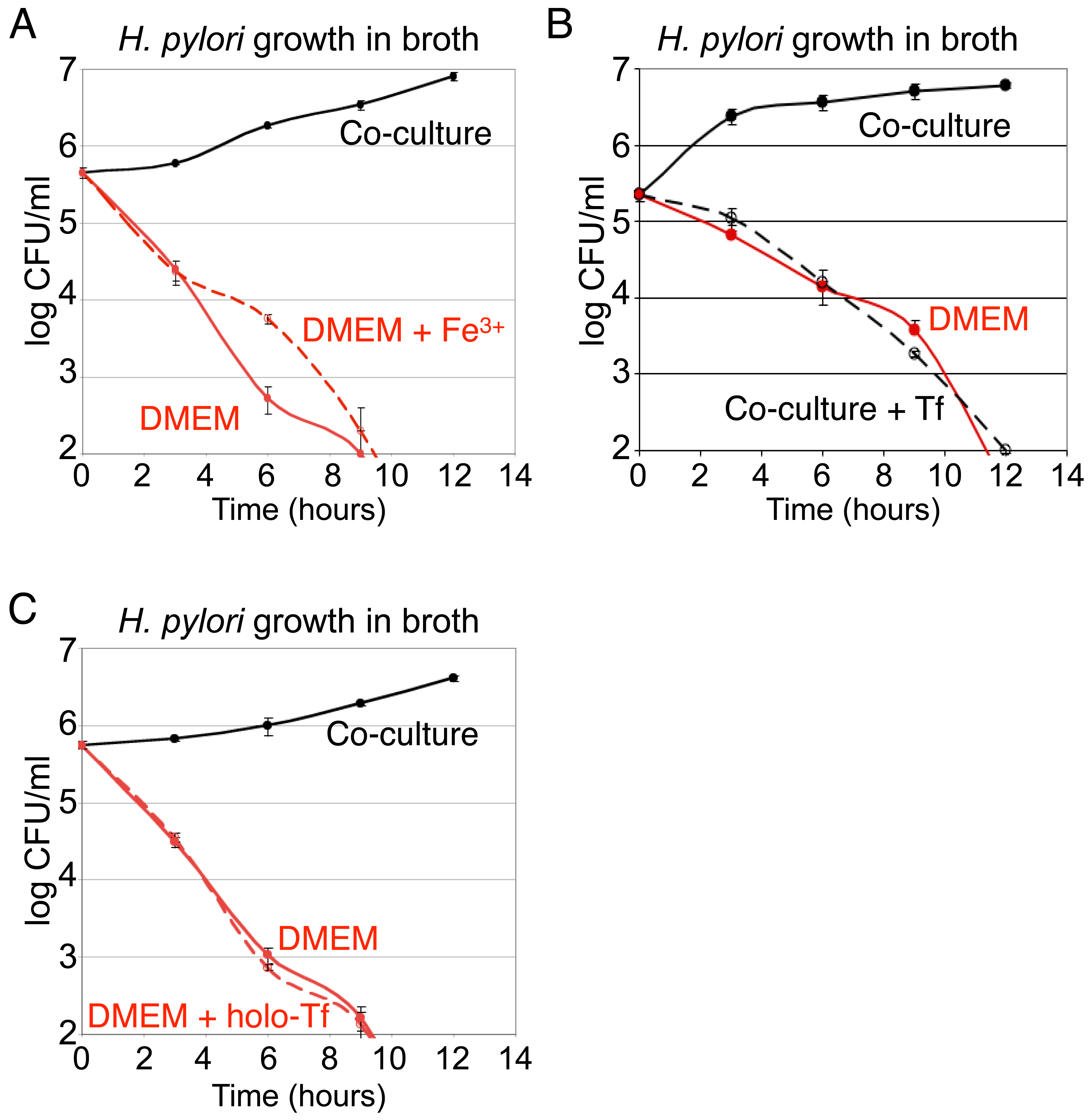

Supplement: Figure S1 — Addition of iron to DMEM is not sufficient to support Hp growth in the absence of host cells. (A) Addition of iron to DMEM is not sufficient to support Hp growth in liquid culture. Plate-grown WT was used to inoculate co-culture media (solid black line), DMEM (red line) or DMEM containing 100 µM ferric chloride (Fe3+; dashed red line), in the absence of host cells. Samples were taken over time and plated for CFU counts. (B) Partially saturated transferrin can inhibit Hp growth in broth. Plate-grown WT was used to inoculate co-culture media (solid black line), DMEM (red line) or co-culture media containing 75 µg/ml partially saturated transferrin (Tf; dashed black line), in the absence of host cells. Samples were taken over time and plated for CFU counts. (C) Addition of holotransferrin to DMEM is not sufficient to support Hp growth in liquid culture. Plate-grown WT was used to inoculate co-culture media (solid black line), DMEM (red line) or DMEM containing 75 µg/ml holotransferrin (holotf; dashed red line), in the absence of host cells. Samples were taken over time and plated for CFU counts. (TIF) [file ppat.1002050.s001.tif]

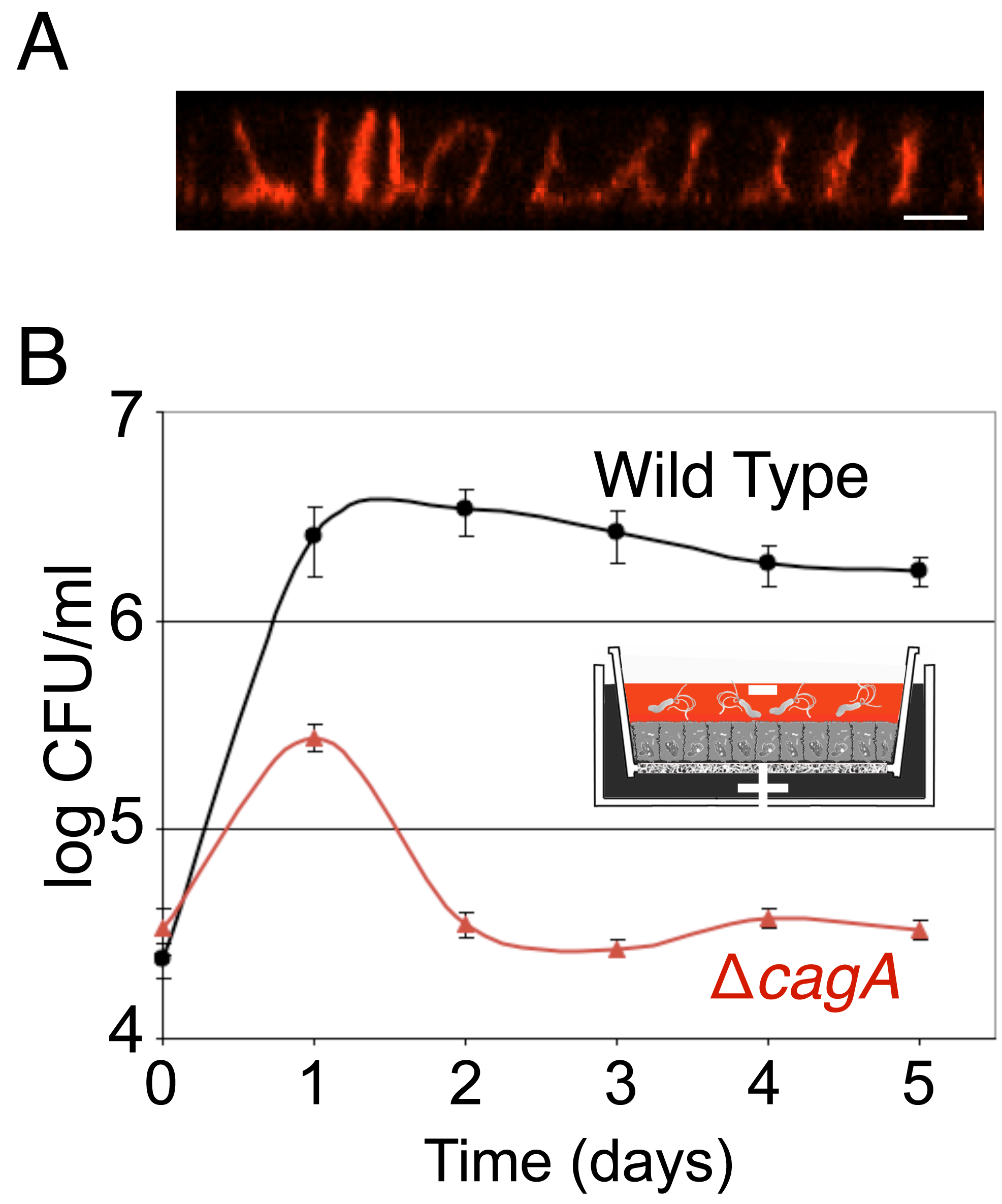

Supplement: Figure S2 — Characterization of MDCK cells stably expressing human transferrin receptor. (A) Basolateral localization of transferrin receptor. Fluorescent transferrin (red) was added to the basal chamber of a polarized monolayer of MDCK cells stably expressing human transferrin receptor, and incubated for 30 minutes on ice before fixation. A cross section through the monolayer is shown. Scale bar 10 µm. (B) MDCK cells stably expressing human transferrin receptor were polarized on Transwell filters and infected with WT or ΔcagA. Co-culture media (+) was added basally and DMEM (−) added apically. Samples were taken daily from the apical chamber and plated for CFU counts. (TIF) [file ppat.1002050.s002.tif]

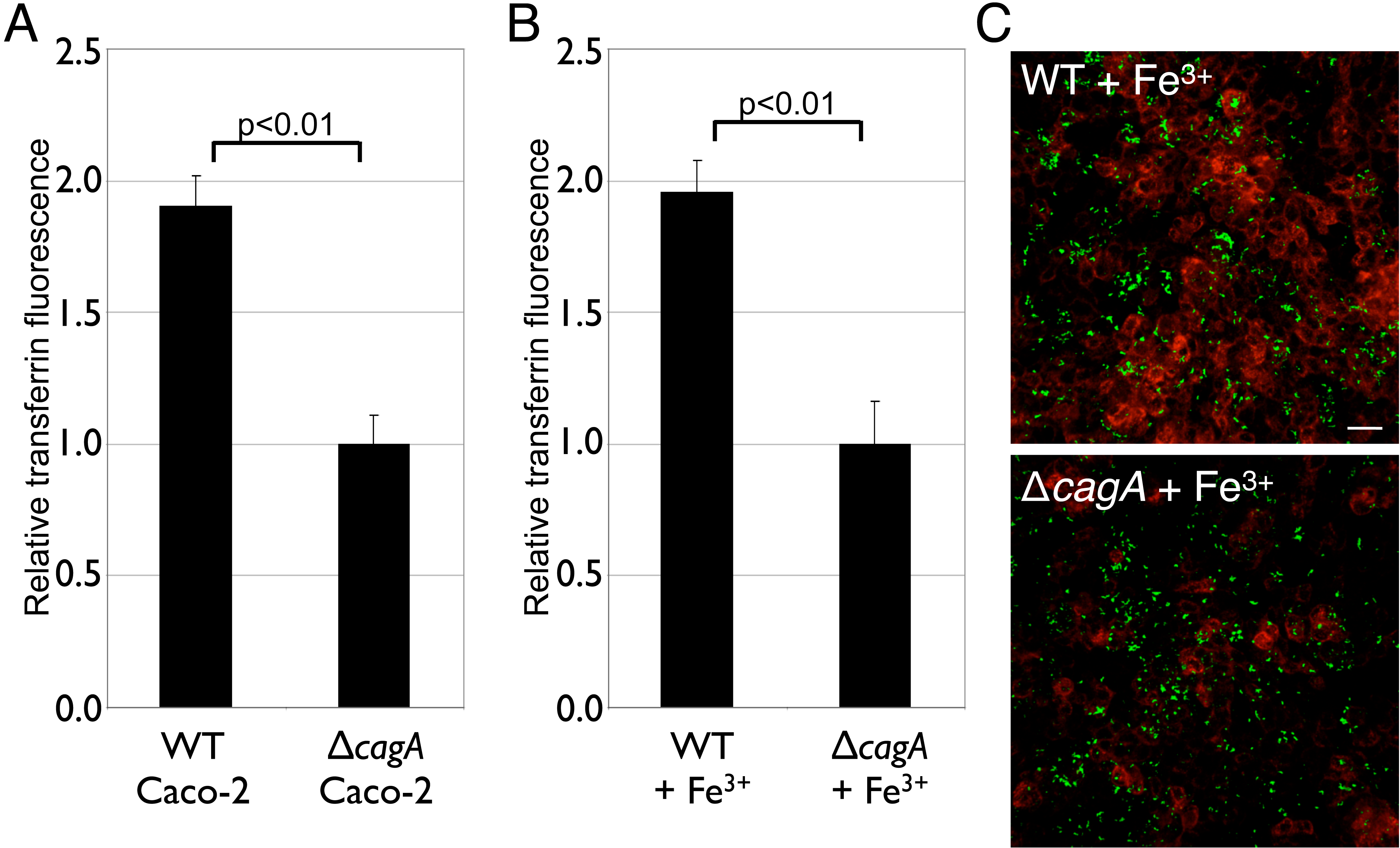

Supplement: Figure S3 — CagA-dependent increase of internalized transferrin occurs in multiple polarized epithelial lines. (A) Hp colonization affects Caco-2 cell transferrin internalization. Polarized Caco-2 cells were infected for 18 hours with WT or ΔcagA. Fluorescent transferrin was added to the basal chamber and incubated on ice for 30 minutes, unbound transferrin washed away, then further incubated for 30 minutes at 37°C to allow uptake of bound transferrin. The graph shows quantitative data of the transferrin fluorescence signal at 30 minutes post-uptake, determined from multiple 3D confocal images. p-value was obtained with a Mann-Whitney statistical test. (B and C) Differences in WT vs. ΔcagA effects on host cell transferrin internalization are not due to differences in bacterial numbers. Polarized MDCK cells stably expressing human transferrin receptor in the Transwell system, with 100 µM ferric chloride added to the apical chamber, were infected for 2 days with WT or ΔcagA. Fluorescent transferrin uptake assay was carried out as in (A). The transferrin fluorescence signal was quantified at 30 minutes post-uptake (B). p-value was obtained with a Mann-Whitney statistical test. 3D confocal images of the monolayers at 30 minutes post-uptake of fluorescent transferrin (red) are shown in (C). Bacteria are visualized with anti-Hp antibodies (green). Scale bar 20 µm. (TIF) [file ppat.1002050.s003.tif]

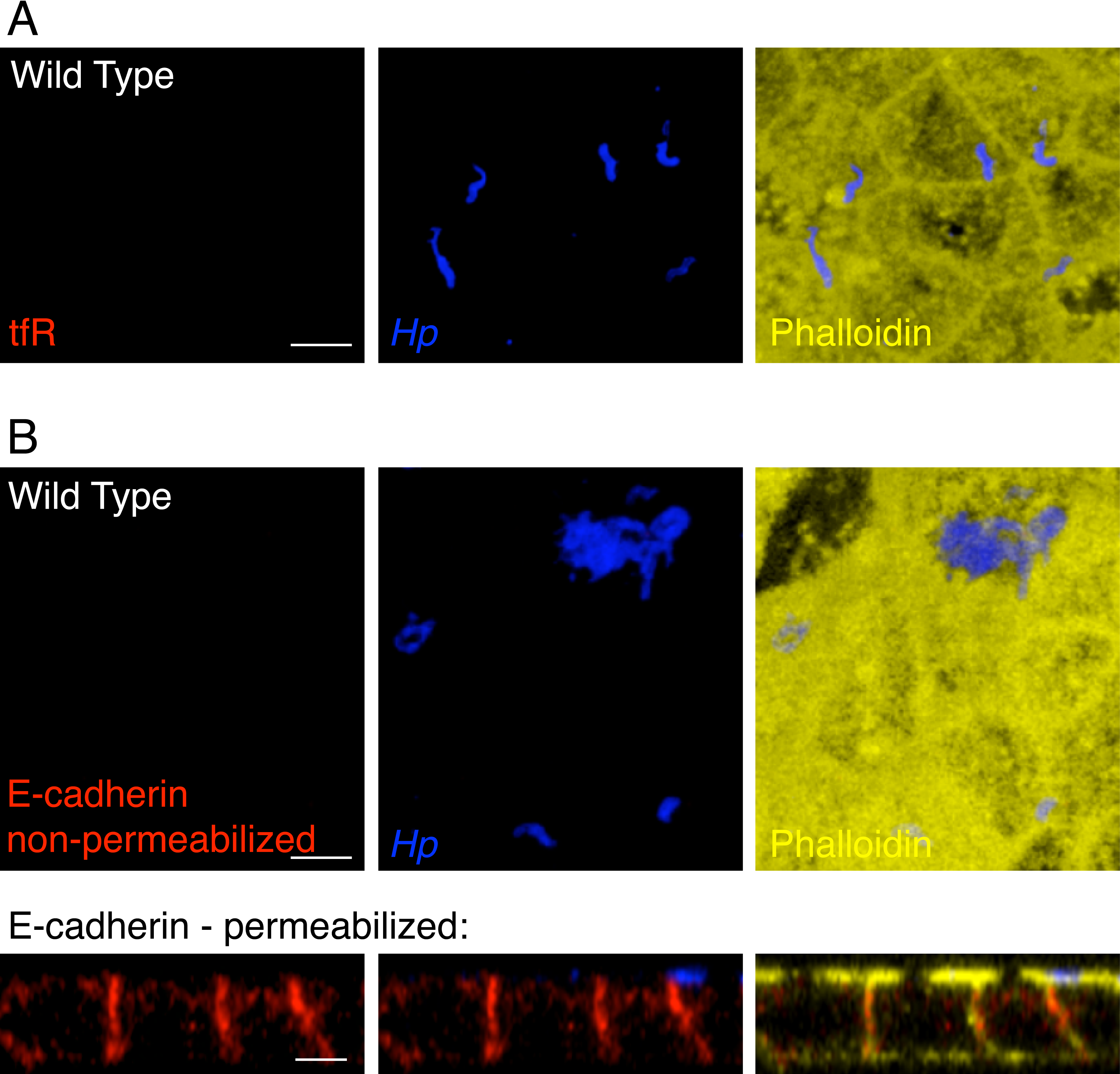

Supplement: Figure S4 — Mislocalization of transferrin receptor to sites of bacterial microcolonies is specific. (A) Initial attachment of Hp to host cells does not result in transferrin receptor mislocalization. Polarized MDCK cells in the Transwell system were infected with WT for 5 minutes and fixed immediately. Apical staining with anti-transferrin receptor antibodies was carried out on non-permeabilized samples. Bacteria are visualized with anti-Hp antibodies (blue), transferrin receptor (tfR) is stained red, and phalloidin staining of f-actin is shown in yellow. 3D confocal images are shown. Scale bar 5 µm. (B) Not all basolateral proteins are mislocalized to bacterial microcolonies. Polarized MDCK cells on Transwell filters were infected with WT for 2 days, then fixed and stained with antibodies against E-cadherin (red) either from the apical surface without permeabilization (top panels), or with permeabilization (bottom panels, cross-section). Bacteria are stained with anti-Hp antibodies (blue), and phalloidin staining of f-actin is shown in yellow. Scale bars 5 µm. (TIF) [file ppat.1002050.s004.tif]

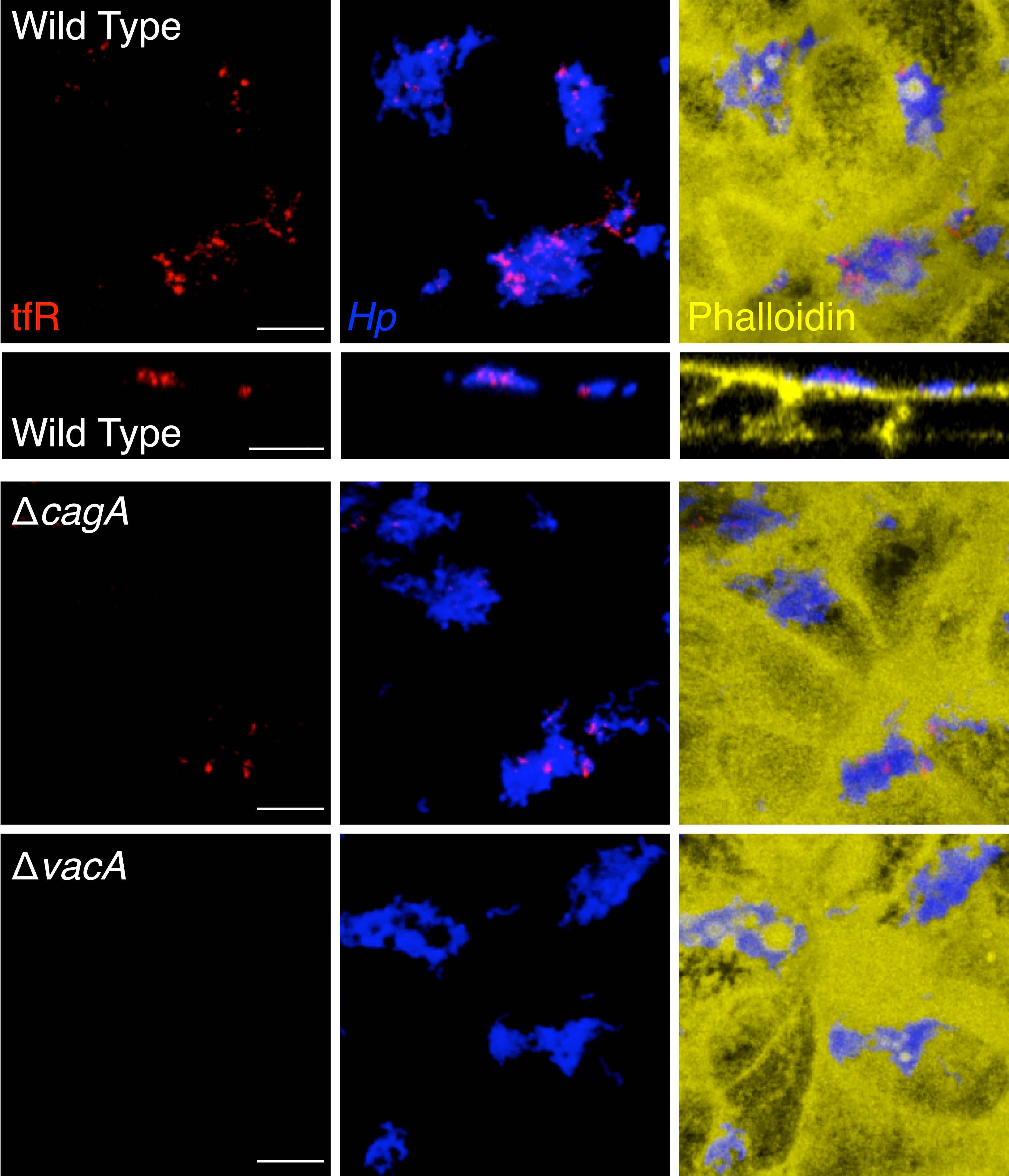

Supplement: Figure S5 — Apical mislocalization of transferrin receptor to sites of bacterial microcolonies occurs in multiple cell types. Polarized Caco-2 cells in the Transwell system were infected with WT, ΔcagA, or ΔvacA for 18 hours. Apical staining with anti-transferrin receptor antibodies was carried out on non-permeabilized samples. Bacteria are visualized with anti-Hp antibodies (blue), transferrin receptor (tfR) is stained red, and phalloidin staining of f-actin is shown in yellow. 3D confocal images are shown, and cross-sectional view is also presented for WT (second row). Scale bars 5 µm. (TIF) [file ppat.1002050.s005.tif]

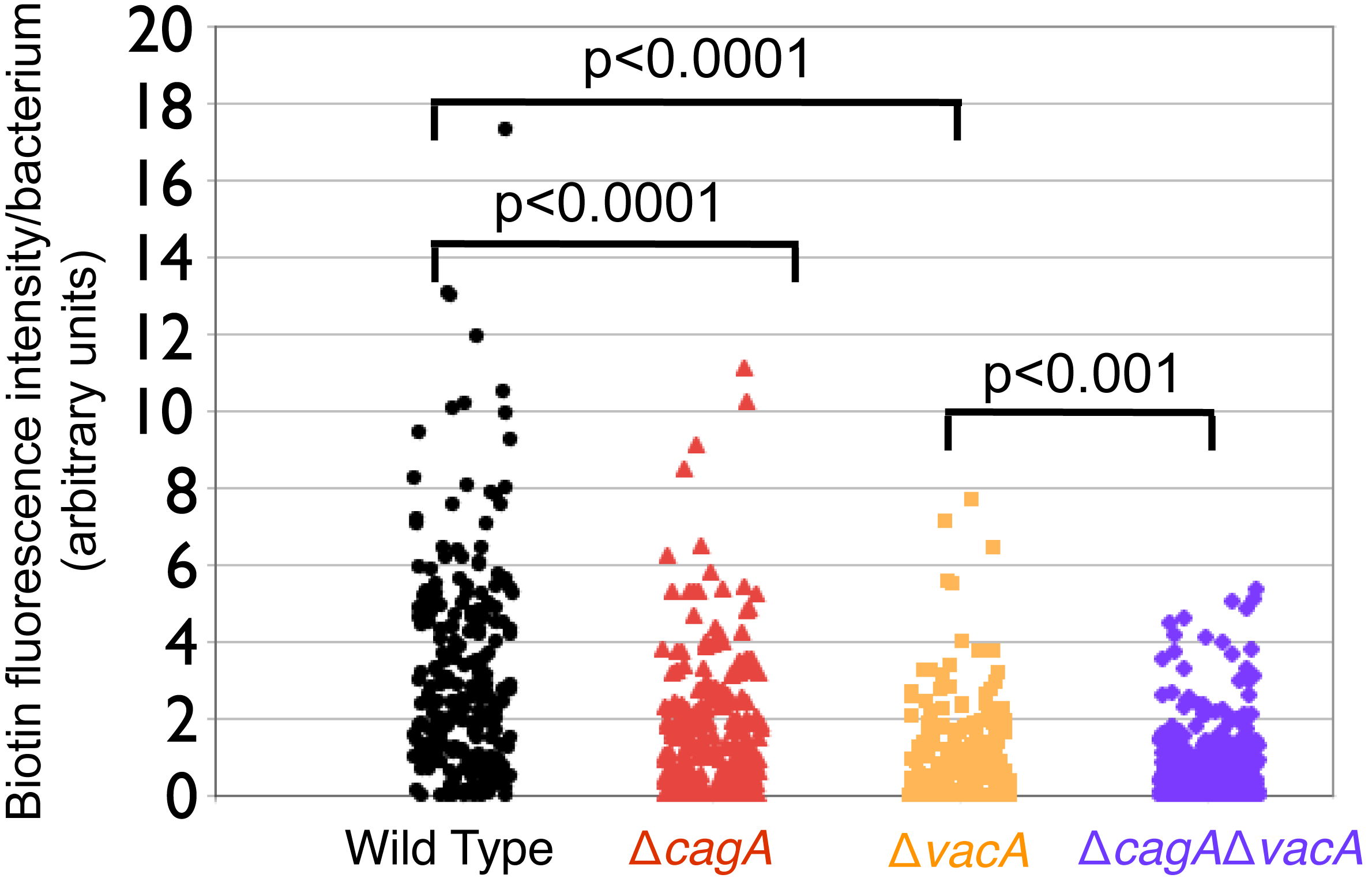

Supplement: Figure S6 — CagA and VacA both contribute to mislocalization of basolateral proteins to the apical surface at sites of bacterial attachment. Polarized cells infected apically with WT, ΔcagA, ΔvacA, or ΔcagAΔvacA for 2 days were selectively biotinylated on ice at the basolateral surface, then incubated for 30 minutes at 37°C before fixation and apical streptavidin staining. Each point represents the total fluorescence intensity of apically-exposed biotin associated with a microcolony, divided by the number of bacteria present in that microcolony. p-values were obtained with a Mann-Whitney statistical test. (TIF) [file ppat.1002050.s006.tif]

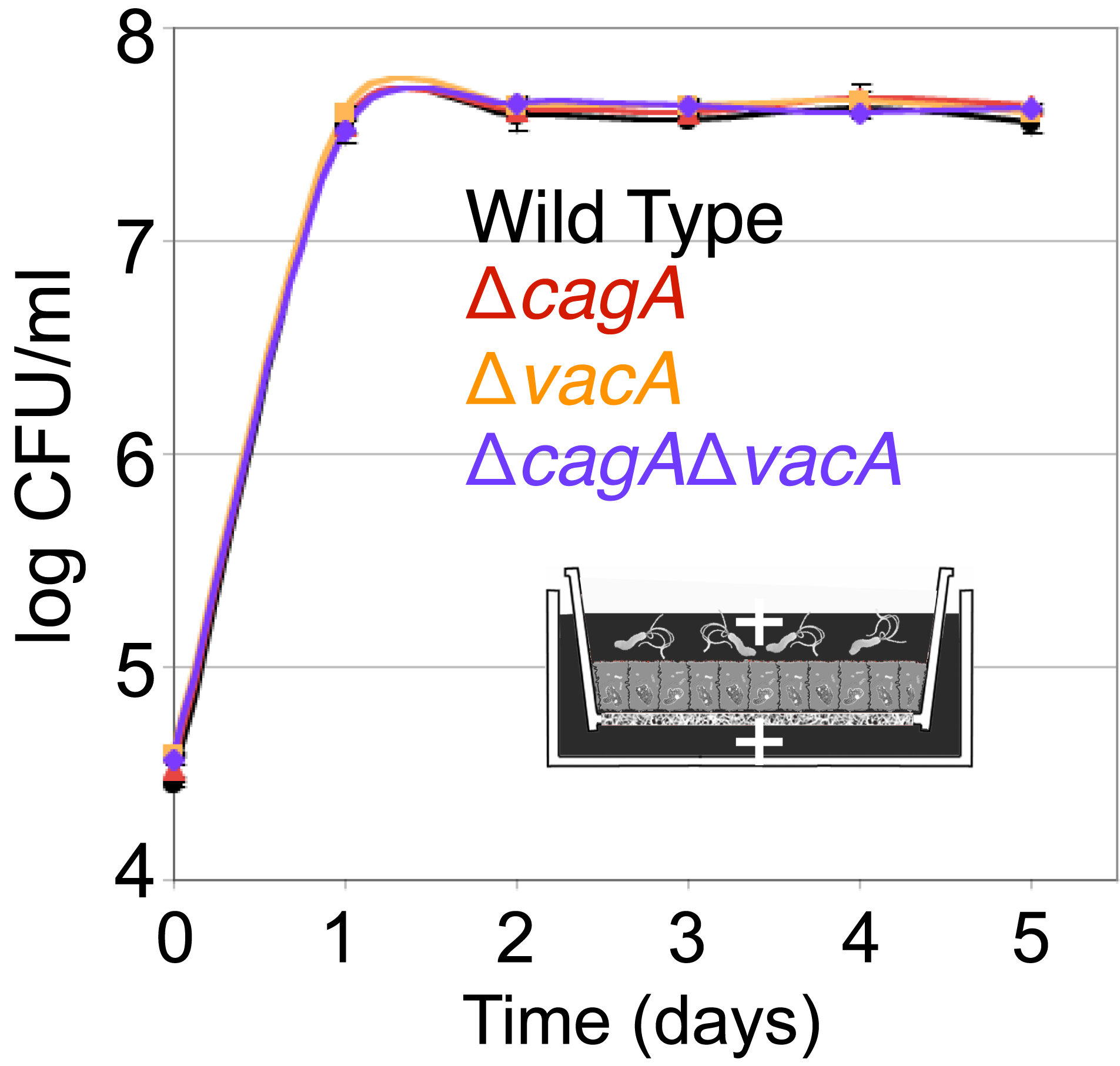

Supplement: Figure S7 — ΔcagA, ΔvacA, and ΔcagAΔvacA mutants grow as well as WT in the presence of nutrients. Polarized MDCK cells in the Transwell system were infected with WT, ΔcagA, ΔvacA, or ΔcagAΔvacA. Co-culture media (+) was added both apically and basally. Samples were taken daily from the apical chamber and plated for CFU counts. (TIF) [file ppat.1002050.s007.tif]

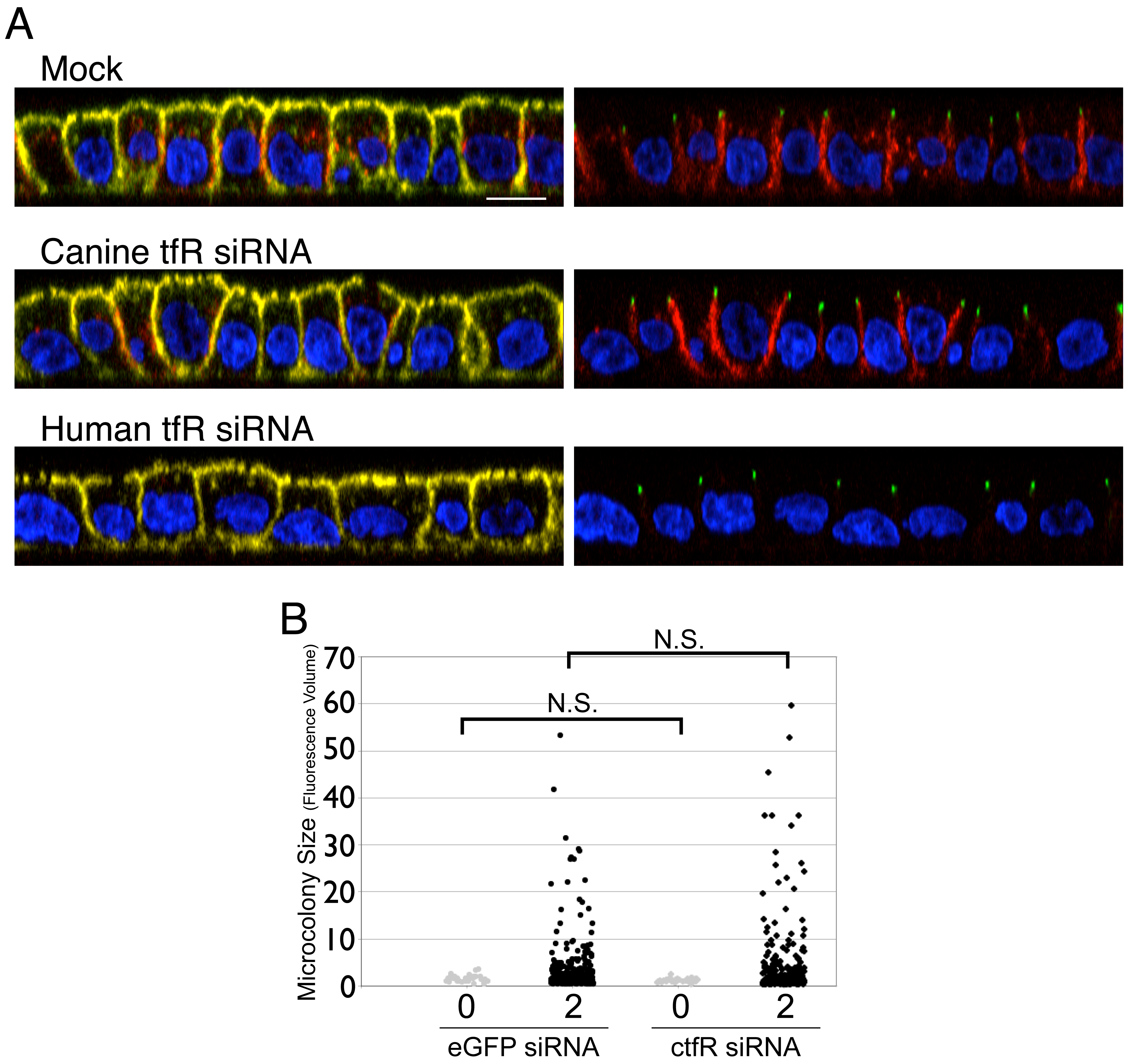

Supplement: Figure S8 — Effect of canine transferrin receptor knockdown is specific. (A) siRNA against canine transferrin receptor is specific. MDCK cells stably expressing human transferrin receptor were mock transfected, or transfected with siRNA against canine or human transferrin receptor (tfR). After polarization, fluorescent human transferrin (red) was added to the basal chamber, and incubated for 30 minutes on ice before fixation. Cross sections through the monolayers are shown. Nuclei are stained with DAPI (blue), phalloidin staining of f-actin is shown in yellow, and cellular tight junctions are visualized with anti-ZO-1 (green). Scale bar 10 µm. (B) Quantification of Hp microcolony sizes on MDCK cells stably expressing human transferrin receptor, transfected with siRNAs directed against canine transferrin receptor (ctfR) or eGFP as a control. Data from 0 and 2 days post-infection are shown. Microcolony sizes were determined from multiple 3D confocal images. Each point on the graph represents a microcolony. p-values were obtained with a Mann-Whitney statistical test. N.S. indicates no statistical significance. (TIF) [file ppat.1002050.s008.tif]

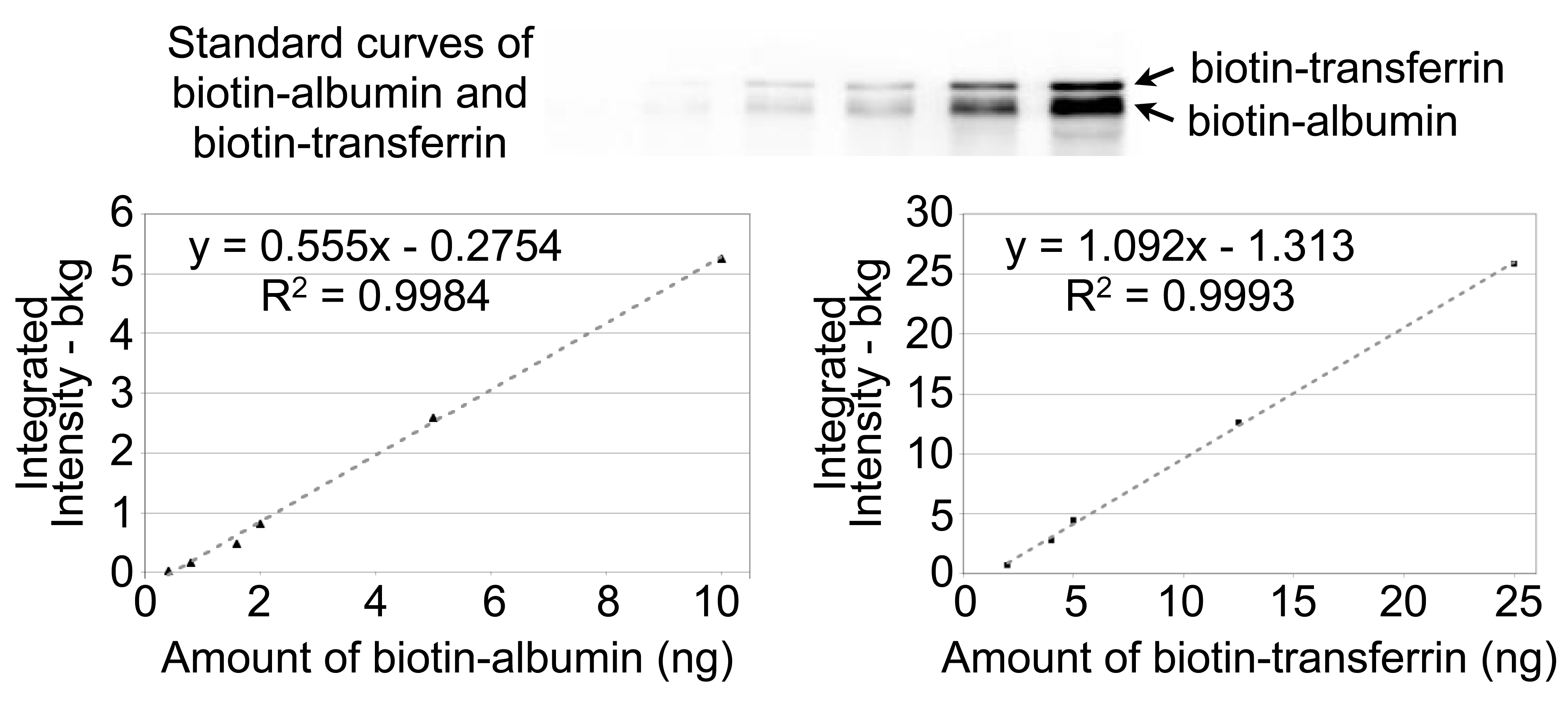

Supplement: Figure S9 — Detection limit and linear range of biotin-albumin and biotin-transferrin measurements. Samples of co-culture media containing biotin-albumin (range 0.4 ng to 10 ng) and biotin-transferrin (range 1 ng to 25 ng) were loaded and separated by SDS-PAGE and transferred to a nitrocellulose membrane. The membrane was probed with Alexa-fluor 647-conjugated streptavidin and bands visualized by the LI-COR Odyssey Scanner and quantified. Arbitrary units were used for the integrated intensity graph. The data was plotted as integrated intensity minus the background (bkg). Best-fit linear curves for the data are shown, as are the linear formulas and their fit. (TIF) [file ppat.1002050.s009.tif]

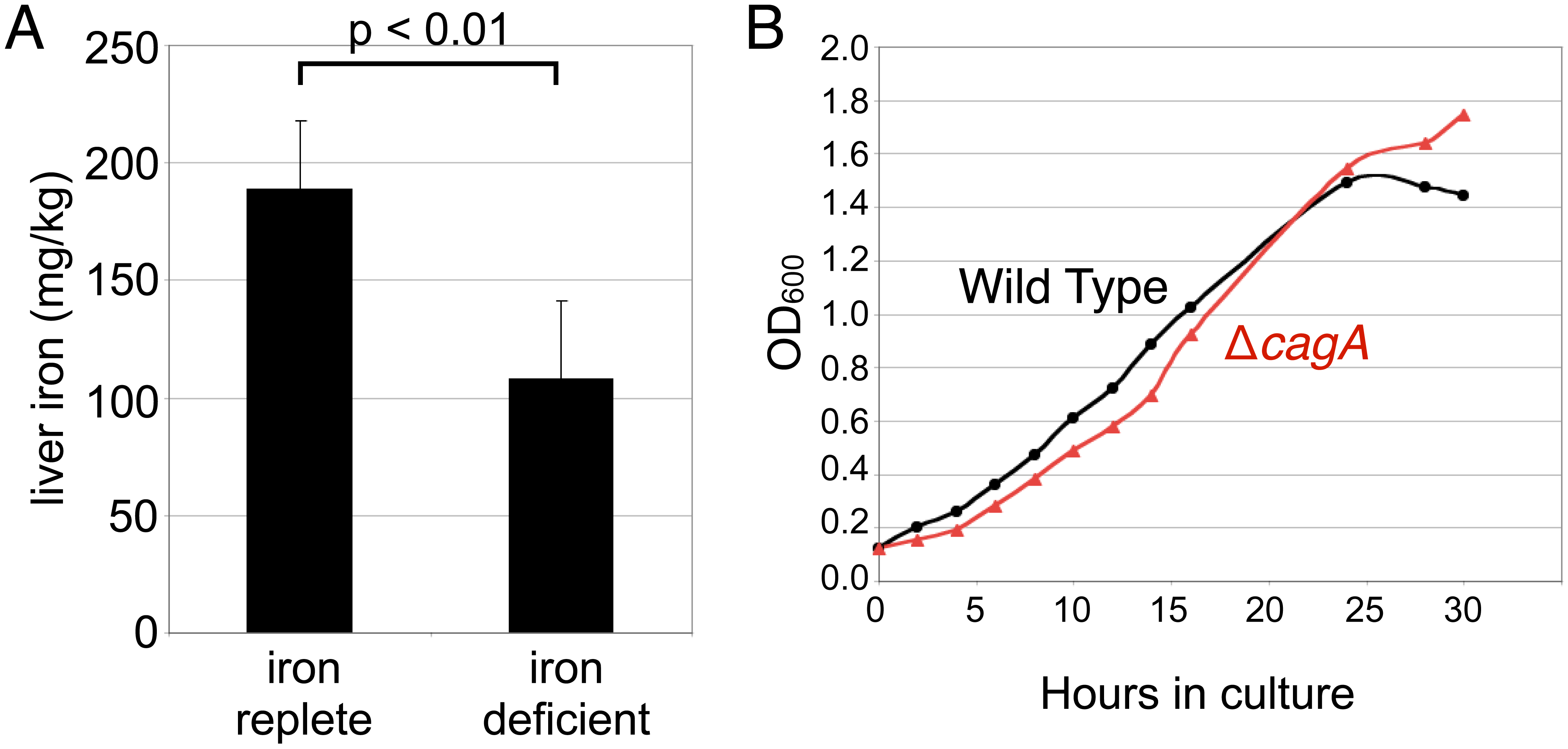

Supplement: Figure S10 — Iron depletion of Mongolian gerbils and in vitro growth curves of Hp strain 7.13 WT and its isogenic ΔcagA mutant. (A) Dietary iron restriction leads to decreased iron levels. Mongolian gerbils were maintained on a regular, iron-replete diet, or on an iron-deficient diet for 3 weeks prior to WT Hp infection, and throughout the course of the 6-week infection. Liver samples were analyzed by inductively coupled plasma-mass spectrometry (Applied Speciation and Consulting, LLC). p-values were obtained with a Mann-Whitney statistical test. (B) Hp strain 7.13 WT and its isogenic ΔcagA mutant grow equally well in nutrient-rich broth. Growth of Hp strain 7.13 WT and ΔcagA in Brucella broth + 10% FBS was followed over 30 hours by measurement of optical density at 600 nm (OD600). Similar results were obtained in 3 independent experiments. (TIF) [file ppat.1002050.s010.tif]

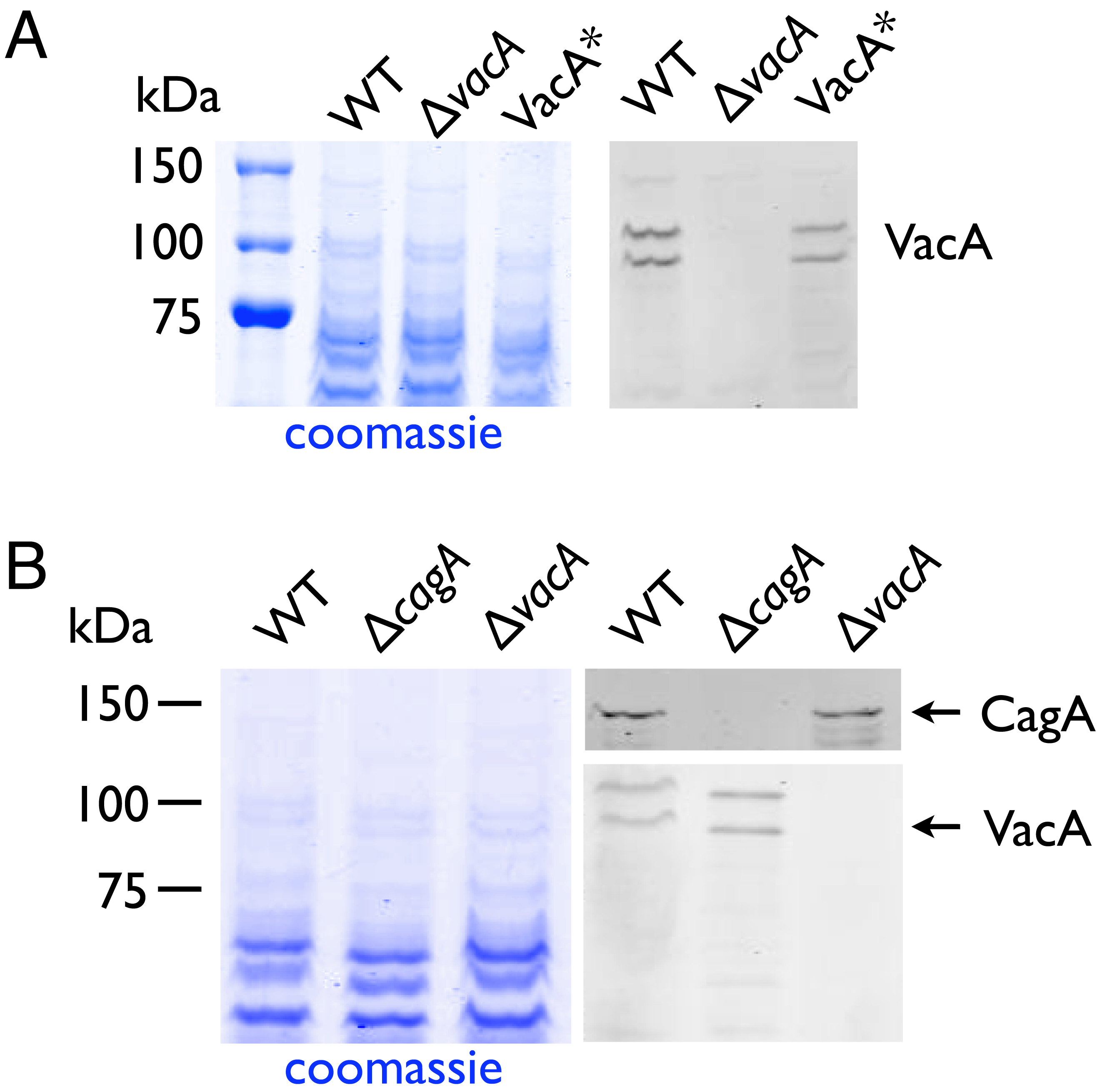

Supplement: Figure S11 — Verification of mutants by immunoblot. (A) Complementation of ΔvacA restores VacA expression. Lysates of Hp grown on Columbia blood agar plates were separated by SDS-PAGE, then either stained with Coomassie Blue or transferred to a nitrocellulose membrane and immunoblotted with polyclonal antibodies against VacA. VacA* is the complemented ΔvacA mutant. (B) CagA and VacA expression is not affected by deletion of vacA and cagA respectively. Lysates of Hp grown on Columbia blood agar plates were separated by SDS-PAGE, then either stained with Coomassie Blue or transferred to a nitrocellulose membrane and immunoblotted with polyclonal antibodies against CagA or VacA. (TIF) [file ppat.1002050.s011.tif]

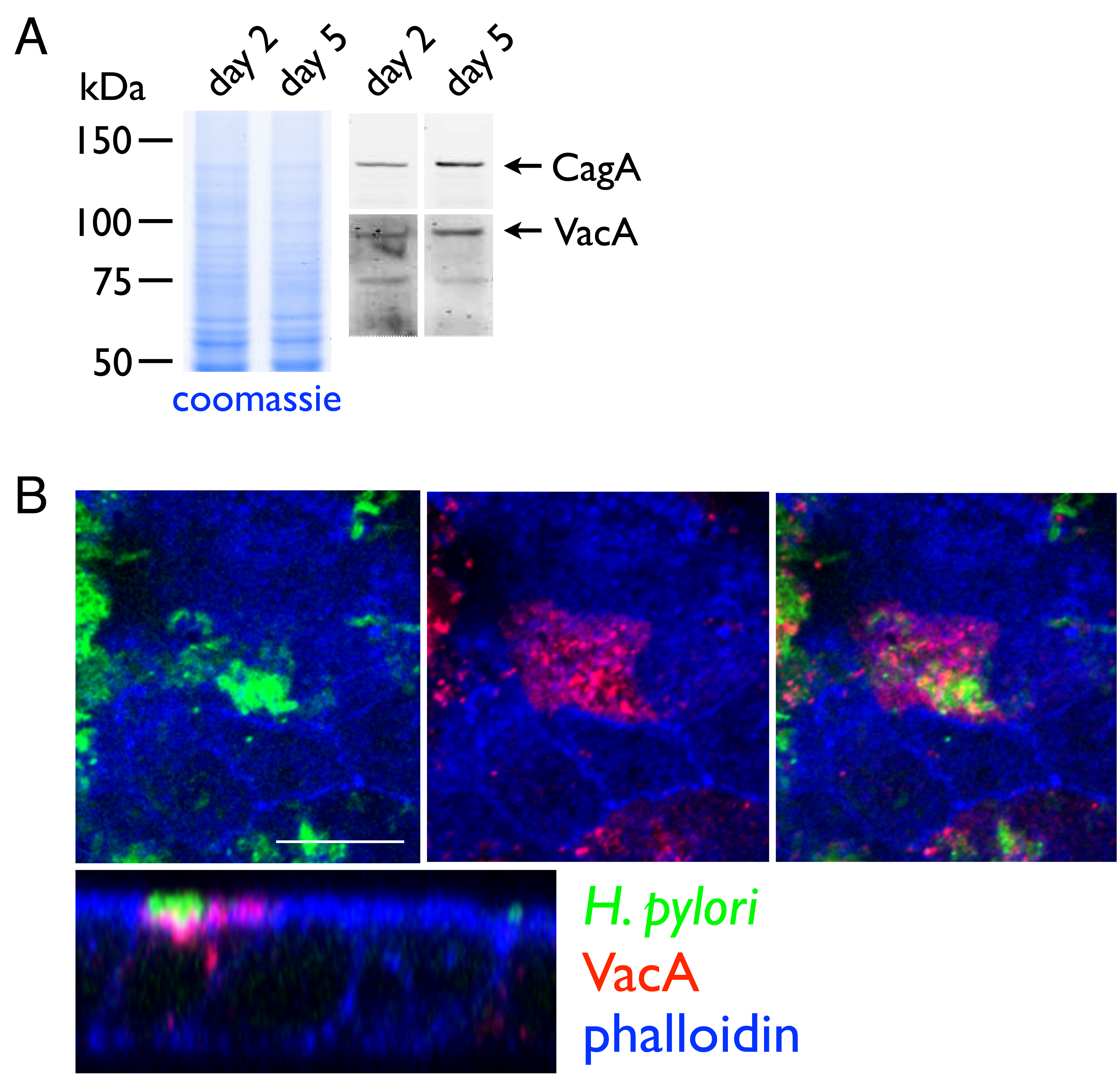

Supplement: Figure S12 — CagA and VacA expression by Hp during infection in the Transwell system. (A) Hp colonizing the polarized epithelium express CagA and VacA. Polarized MDCK cells in the Transwell system were infected with WT. Free-swimming bacteria were washed away with DMEM and lysates of infected cells were collected at 2 or 5 days post-infection, separated by SDS-PAGE, then either stained with Coomassie Blue or transferred to a nitrocellulose membrane and immunoblotted with antibodies against CagA or VacA. (B) Hp colonizing the polarized epithelium deliver VacA into the host cells. Polarized MDCK cells in the Transwell system were infected with WT for 2 days, then fixed and stained with antibodies against Hp (green) and VacA (red). Phalloidin staining of f-actin is shown in blue. Scale bar 10 µm. (TIF) [file ppat.1002050.s012.tif]
